# Unravelling hybridization in *Phytophthora* using phylogenomics and genome size estimation

**DOI:** 10.1186/s43008-021-00068-w

**Published:** 2021-07-01

**Authors:** Kris Van Poucke, Annelies Haegeman, Thomas Goedefroit, Fran Focquet, Leen Leus, Marília Horta Jung, Corina Nave, Miguel Angel Redondo, Claude Husson, Kaloyan Kostov, Aneta Lyubenova, Petya Christova, Anne Chandelier, Slavcho Slavov, Arthur de Cock, Peter Bonants, Sabine Werres, Jonàs Oliva Palau, Benoit Marçais, Thomas Jung, Jan Stenlid, Tom Ruttink, Kurt Heungens

**Affiliations:** 1 Flanders Research Institute for Agriculture, Fisheries and Food (ILVO), Plant Sciences Unit, Burg. Van Gansberghelaan 96, 9820 Merelbeke, Belgium; 2 Phytophthora Research Centre (PRC), Mendel University, 613 00 Brno, Czech Republic; 3 Laboratory of Molecular Biotechnology and Phytopathology, Centre for Mediterranean Bioresources and Food, University of Algarve, 8005–130 Faro, Portugal; 4 Julius Kühn Institute (JKI) – Federal Research Center for Cultivated Plants, Institute for Plant Protection in Horticulture and Forestry (GF), Messeweg 11/12, 38104 Braunschweig, Germany; 5 Department of Forest Mycology and Plant Pathology, Swedish University of Agricultural Sciences (SLU), Box 7026, 750 07 Uppsala, Sweden; 6 Ministère de l’agriculture et de l’alimentation, Direction générale de l’alimentation (DGAL), Sous Direction de la Qualité et de la Protection des Végétaux (SDQPV), Département de la Santé des Forêts, 75015 Paris, France; 7 AgroBioInstitute (ABI), Agricultural Academy, 8 Dragan Tsankov blvd., 1164 Sofia, Bulgaria; 8 Life Sciences Department, Walloon Agricultural Research Centre (CRAW), Rue de Liroux 4, 5030 Gembloux, Belgium; 9 Westerdijk Fungal Biodiversity Institute, P.O. Box 85167, 3508 AD Utrecht, the Netherlands; 10 Wageningen University & Research, Business Unit Biointeractions & Plant Health, PO BOX 16, 6700 AA Wageningen, the Netherlands; 11 Department of Crop and Forest Sciences, Agrotecnio Center CERCA, University of Lleida, Alcalde Rovira Roure 191, 25198 Lleida, Spain; 12 Université de Lorraine – Institut National de la Recherche Agronomique (INRAe), L’Unité Mixte de Recherche Interactions arbres/microorganismes (UMR IAM), 54000 Nancy, France

**Keywords:** Flow cytometry, GBS, Oomycete, Hybrid, Phylogeny, Polyploidy

## Abstract

The genus *Phytophthora* comprises many economically and ecologically important plant pathogens. Hybrid species have previously been identified in at least six of the 12 phylogenetic clades. These hybrids can potentially infect a wider host range and display enhanced vigour compared to their progenitors. *Phytophthora* hybrids therefore pose a serious threat to agriculture as well as to natural ecosystems. Early and correct identification of hybrids is therefore essential for adequate plant protection but this is hampered by the limitations of morphological and traditional molecular methods. Identification of hybrids is also important in evolutionary studies as the positioning of hybrids in a phylogenetic tree can lead to suboptimal topologies. To improve the identification of hybrids we have combined genotyping-by-sequencing (GBS) and genome size estimation on a genus-wide collection of 614 *Phytophthora* isolates. Analyses based on locus- and allele counts and especially on the combination of species-specific loci and genome size estimations allowed us to confirm and characterize 27 previously described hybrid species and discover 16 new hybrid species. Our method was also valuable for species identification at an unprecedented resolution and further allowed correct naming of misidentified isolates. We used both a concatenation- and a coalescent-based phylogenomic method to construct a reliable phylogeny using the GBS data of 140 non-hybrid *Phytophthora* isolates. Hybrid species were subsequently connected to their progenitors in this phylogenetic tree. In this study we demonstrate the application of two validated techniques (GBS and flow cytometry) for relatively low cost but high resolution identification of hybrids and their phylogenetic relations.

**Supplementary Information:**

The online version contains supplementary material available at 10.1186/s43008-021-00068-w.

## INTRODUCTION

The genus *Phytophthora* is an important genus of plant pathogenic oomycetes. These organisms were long classified as fungi because of similarities in life-style and morphology, but they differ in cytological, biochemical, and genomic aspects. Moreover, oomycetes are diploid, in contrast with the primarily monoploid fungi (Beakes et al. 2012). Oomycetes are now classified in the diverse *Straminipila* lineage within the *Straminipila-Alveolata-Rhizaria* (SAR) eukaryotic supergroup (McCarthy and Fitzpatrick 2017). Important *Phytophthora* species in agricultural as well as natural plant ecosystems include *P. infestans*, *P. sojae, P. ramorum,* and *P. cinnamomi* (Fry 2008; Grünwald et al. 2012; Hardham 2005; Jung et al. 2018b; Tyler 2007). Known *Phytophthora* species are classified in 12 phylogenetic clades, some of which are subdivided into rather diverse subclades (Jung et al. 2017b; Yang et al. 2017). Species identification and phylogenetic classification is typically based on DNA sequence information from specific loci (Blair et al. 2008; Cooke et al. 2000; Kroon et al. 2004; Martin et al. 2014; Yang et al. 2017), although the discriminative power of some of these markers within subclades is limited (Yang and Hong 2018).

Application of these molecular identification techniques combined with an increasing number of surveys has led to the description of many new *Phytophthora* species in the last 25 years (Yang et al. 2017). Together with the identification of novel species came the relatively recent discovery of several *Phytophthora* hybrids. So far, natural hybrids have been found in clade 1 (*P. andina* (Goss et al. 2011a), *P.* ×*serendipita,* and *P.* ×*pelgrandis* (Man in ‘t Veld et al. 2012)); clade 6 (*P.* ×*stagnum* (Yang et al. 2014) and other hybrids (Burgess 2015; Jung et al. 2018a; Nagel et al. 2013)); and clade 7 (*P.* ×*alni*, *P.* ×*multiformis* (Brasier et al. 1999, 2004; Husson et al. 2015; Ioos et al. 2006), *P.* ×*cambivora* and other hybrids (Jung et al. 2017c)). Also in clade 8 (Bertier et al. 2013; Safaiefarahani et al. 2016), clade 2 and clade 9 (Jung et al. 2017a) several unnamed or informally designated hybrids have been discovered. Clade 2 species *P. meadii* has also been suspected to be a hybrid (Sansome et al. 1991). In addition, sexual and somatic hybrids have been created under laboratory conditions, even between species from different clades (Érsek and Nagy 2008), but natural interclade hybrids have not been reported previously.

Hybridization and polyploidization not only play a key role in the evolution of plants and animals (Mallet 2007; Soltis 2013; Soltis and Soltis 2009; Van de Peer et al. 2017), but also in the evolution of fungi and oomycetes (Bertier et al. 2013; Brasier 2001; Callaghan and Guest 2015; Schardl and Craven 2003). In plants and animals, hybridization may result in increased vigour compared to parental species (Abbott et al. 2013). In addition, polyploidy plays a key role in bursts of adaptive speciation (Alix et al. 2017). Moreover, hybridization (Chown et al. 2015; Ellstrand and Schierenbeck 2000; Schierenbeck and Ellstrand 2009) and polyploidization (Pandit et al. 2011; te Beest et al. 2012) are thought to be important factors that affect invasiveness of species. This also seems to be true in *Phytophthora*, where ploidy levels can change (e.g. several polyploid lineages in *P. infestans*; Catal et al. 2010; Yoshida et al. 2013; Li et al. 2017) and where hybrids such as *P.* ×*serendipita* and *P.* ×*alni* seem to outcompete their parental species (Ioos et al. 2006; Man in ‘t Veld et al. 2007).

Host specialization can be a barrier that prevents native *Phytophthora* species from hybridizing (Giraud et al. 2006). In addition, reproductive barriers prevent sympatric species from crossing. Allopatric species, on the other hand, are considered to be more prone to hybridization when they come into contact with each other (Beckerman et al. 2014; Stukenbrock and McDonald 2008). Human activity, with plant trade in particular, can bring allopatric *Phytophthora* species into contact (Goss et al. 2011b; Jung et al. 2016; Jung et al. 2018b; Liebhold et al. 2012; Redondo et al. 2018a; Santini et al. 2013). These introduced species will likely have strong effects if they infect native host plants because they lack a co-evolutionary history with those hosts. This, in turn, increases the chance of interspecific mating between the native and the introduced *Phytophthora* species, which might present a serious threat to biosecurity (Beckerman et al. 2014; Callaghan and Guest 2015). Early detection and characterization of invasive *Phytophthora* species and especially of hybrids is therefore essential in the prevention or containment of disease outbreaks (Keriö et al. 2019).

*Phytophthora* hybrids are usually identified via cytology, isozyme analysis and/or sequencing of one or multiple loci (see above for examples). Polyploid hybrids can also be detected via genome size measurement because such hybrids contain the combined genomes of their parental species (Bertier et al. 2013; Husson et al. 2015). However, large genome sizes can also be the result of autopolyploidization, as observed in *P. infestans* (Li et al. 2017; Martens and Van de Peer 2010). Unambiguous detection of polyploid hybrids thus requires additional techniques. For example, polyploid hybrids can be recognized by the presence of more than two clearly distinct alleles in a set of reference genes. For reliable detection, this type of analysis requires PCR amplification, cloning, and sequencing of multiple loci. This costly approach limits its use on a large number of samples. Cost-effective alternatives include reduced-representation library sequencing methods such as genotyping-by-sequencing combined with sample indexing during library preparation, which allows genome-wide fingerprinting and multiplexing of libraries (Elshire et al. 2011; Poland et al. 2012; Zimmer and Wen 2015). Genome-wide genetic fingerprinting with Next Generation Sequencing (NGS) techniques has been applied to numerous plant and animal species (Anderson et al. 2017; Arbizu et al. 2016; Fernández-Mazuecos et al. 2018; Hohenlohe et al. 2013; Hou et al. 2015; Jones et al. 2013; Stetter and Schmid 2017) and recently to *Phytophthora betacei* (Mideros et al. 2018). The abundance of such genome sequence data provides unprecedented genetic resolution that outcompetes the traditional Sanger sequencing technique for species identification and hybrid detection in a large number of samples. This leads to better resolution of phylogenetic relationships as compared to phylogenies based on one or a few genes.

**Fig. 1:**
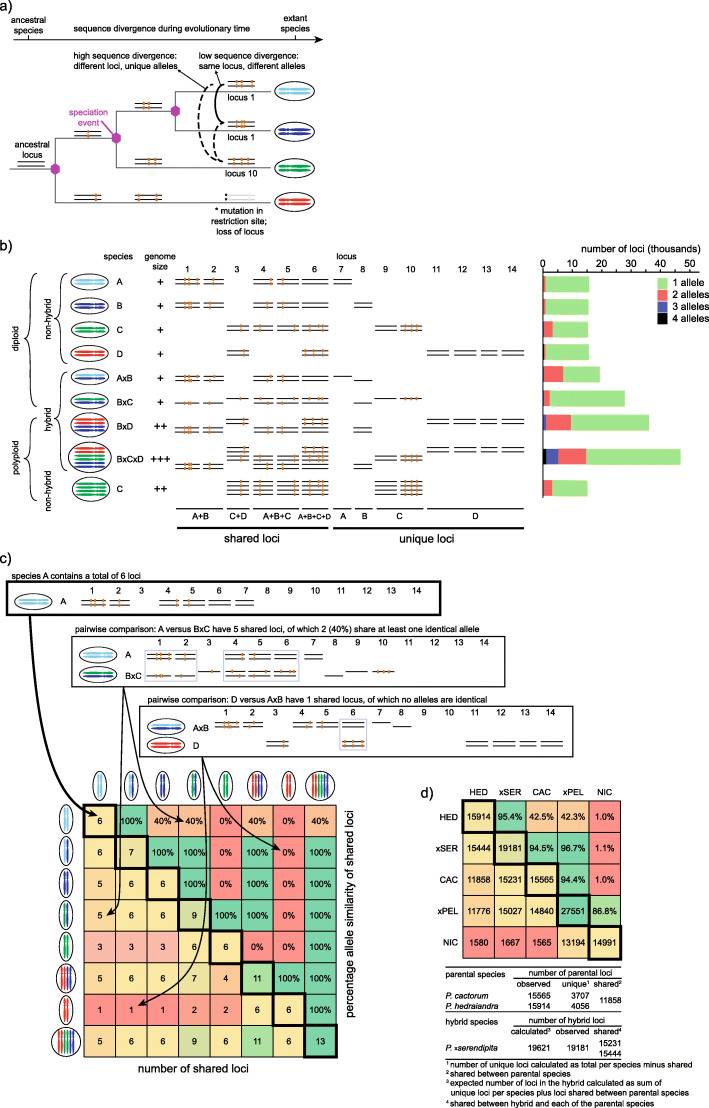
Concept that was used for the identification of *Phytophthora* hybrids using genotyping-by-sequencing (GBS) and flow cytometry. **a**) simplified representation of the evolution of four species and the consequences it has on one of the GBS loci concerning presence/absence of the locus and the SNPs it harbours. For the sake of clarity it does not take other biological processes into account that are important in speciation such as polyploidisation, gene duplication or other genomic rearrangements; **b**) simplified representation of the effect of speciation and hybridization on the genome size, the number of GBS loci and the number of alleles of each locus. The diploid non-hybrid species A-D have a similar number of GBS loci (six in this case). Genetically closely related species are expected to share more loci, while distant species are expected to present more unique loci. The proportion of shared alleles of loci common to two species will also depend on the phylogenetic distance between species (illustrated for loci 9–14). The sexual reproduction system (homo- or heterothallic) will also influence the number of alleles at a given locus. For example, the heterothallic species C will display a larger proportion of biallelic loci than the homothallic species A, B and D. The barplot is based on real numbers (see Table S4); **c**) colour-coded matrix that is the result of the pairwise comparison of the GBS loci as presented in Fig. 1**b**. The number of loci per species is determined (by adding them up horizontally in Fig. 1**b**) and is placed in the diagonal of the matrix. Next, the loci that are shared among each pair of species are scored vertically in Fig. 1**b** and the resulting number is placed in the corresponding cell in the lower triangle of the matrix. Finally, the allele similarity is determined for the shared loci and is placed in the upper triangle of the matrix; **d**) example of the colour-coded matrix that is the result of the pairwise comparison of the loci of *P. cactorum*, *P. hedraiandra*, *P. nicotianae*, *P.* ×*serendipita* and *P.* ×*pelgrandis*. Below the matrix the calculations are shown for *P. cactorum*, *P. hedraiandra*, and their hybrid *P.* ×*serendipita* (see also Table 2)

In this study, we identify and characterize hybrids in the genus *Phytophthora* based on a combination of genome-wide genetic fingerprinting and genome size assessment. During evolution, speciation is associated with accumulating levels of genetic divergence (Fig. 1a). This divergence is reflected in species- or genotype-specific GBS fingerprints in extant species, which consist in the presence (or absence) of loci and in allelic variants at each locus (Fig. 1a and b). Because a hybrid combines its progenitor genomes, it will display a composite fingerprint. On the one hand, a hybrid fingerprint comprises the shared GBS loci of the parental species and their respective unique loci. For this reason, a hybrid typically contains a higher number of loci compared to its progenitors (Fig. 1c). On the other hand, a hybrid fingerprint comprises the combined allelic diversity for shared loci. For this reason, a hybrid typically displays a higher number of alleles per locus compared to its progenitors (Fig. 1c). Polyploid hybrids are further distinguished by their higher number of multi-allelic loci combined with a larger genome size than their progenitors (Fig. 1b).

In the most recent phylogenetic studies on *Phytophthora*, hybrids have typically been treated as “pure” species. Consequently, they are depicted as a single terminal branch, positioned close to one of their progenitor species (Blair et al. 2008; Kroon et al. 2004; Martin et al. 2014; Schardl and Craven 2003; Yang et al. 2017). It is well conceived that reticulate evolutionary events such as hybridization are difficult to fit into a tree-like phylogeny (Kroon et al. 2004) and can cause ambiguous positioning of hybrids in a phylogenetic tree, especially when concerning hybrids of distantly related progenitors (McDade 1992). Hybridization can also lead to artefacts such as “trickle-down discordance” (Baum 2009). By excluding hybrids from primary phylogenetic analyses, and only anchoring them afterwards to the phylogenetic tree, these issues can be circumvented. In addition, application of genome-wide data used for phylogenetic analyses in several genera can prevent the problems that arise in phylogenies based on a limited number of loci that lack sufficient phylogenetic signal (Arbizu et al. 2016; Escudero et al. 2014; Hou et al. 2015; Stetter and Schmid 2017).

The recognition of hybrids plays an important role in phylogenetics, as well as in pest risk assessment, but identification of hybrids is currently difficult. Therefore, the main objective of this study was to develop a new strategy to reliably identify and characterize known and novel *Phytophthora* hybrids. A broad collection of 614 isolates was gathered, representing 132 *Phytophthora* and four outgroup species. This collection was comprised of reference isolates and potential hybrids that were selected based on ITS sequencing and morphological characterization. The entire collection was subjected to GBS and subsequent reference-free data analysis. This generated several critical genome fingerprint features: the absence/presence of GBS loci, the total number of loci per isolate, and the number of allelic variants per locus (Fig. 1b). The second objective was to combine allele count analysis with genome size measurements for the identification of polyploid hybrids (Fig. 1b). The third objective was to improve the *Phytophthora* phylogeny by constructing a genus-wide phylogenetic tree of exclusively non-hybrid species by applying both a concatenation- and a coalescent-based phylogenomics method to the GBS data, followed by anchoring the hybrids to their progenitors.

## MATERIALS AND METHODS

### Isolates and DNA collection

*Phytophthora* isolates were mainly collected using traditional isolation or baiting techniques during surveys in nurseries, forests, and rivers of European countries in the framework of the EU BiodivERsA Resipath project (https://www.biodiversa.org/1052/download). Details of all isolates are provided in Table S1. In tables and figures, species names are sometimes abbreviated with a three letter code (Table 1). Abbreviations followed by “r” are not included in this table; they concern species that are genetically related to the species indicated by the three letter code. Their genetic relationship was determined based either on the GBS results and phylogenomic analyses in this study, or sequencing during pre-screening, or both. The rDNA ITS sequence of the isolates was used for preliminary identification and selection (data not shown). Isolates with heterozygous sites in the ITS sequence received special attention as potential hybrids. Cultures were either immediately used for flow cytometry (FCM) or DNA extraction after hyphal-tipping or were stored at 12 °C in sterile water until further processing. Additional isolates or DNA were obtained from laboratories participating in the EU Resipath project (ILVO, PRC, JKI, SLU, DGAL, UMR IAM, CRAW, ABI; see author affiliations). We also included DNA from isolates from the Westerdijk Institute (Utrecht, The Netherlands; CBS) and Wageningen Plant Research (Wageningen, The Netherlands; PRI). For DNA extraction, isolates were grown in 20% clarified V8 broth for one to two weeks, depending on the growth rate of the isolate. We verified the absence of contaminating organisms via a shake culture to avoid contaminating sequences in the GBS analysis. The mycelium was placed on a Whatman No1 filter paper (Sigma-Aldrich), rinsed thoroughly with sterile water and vacuum-dried. Approximately 100 mg mycelium was placed in a 1.5 ml microcentrifuge tube, frozen in liquid nitrogen, and manually crushed using a micropestle. DNA was extracted using the Nucleospin Plant II kit (Macherey-Nagel) with extraction buffer PL1, according to the manufacturer’s protocol, except that DNA was eluted in 50 μl elution buffer. Some of the DNA samples from previous collections had been prepared with alternative techniques (marked with an asterisk in Table S1, e.g. all CBS and PRI isolates). Additional analysis revealed that the DNA extraction methods had no effect on the quality of the GBS data (see Table S3). DNA concentrations were measured using a Quantus Fluorometer (Promega) and DNA was stored at − 20 °C. A total of 661 samples (including replicates) were used for GBS and/or FCM analysis (indicated in Table S1).

**Table 1:** Abbreviations of species names used in tables and figures

Abbreviation	Species	Abbreviation	Species	Abbreviation	Species
ACE	*Phytophthora acerina*	HYD	*P. hydropathica*	PSY	*P. psychrophila*
ALT	*P. alticola*	IDA	*P. idaei*	QCT	*P. quercetorum*
AMN	*P. amnicola*	ILI	*P. ilicis*	QUI	*P. quininea*
AND	*P. andina*	INF	*P. infestans*	RAM_EU1	*P. ramorum* EU1
ASP	*P. asparagi*	INS	*P. insolita*	RAM_EU2	*P. ramorum* EU2
AUS	*P. austrocedrae*	INU	*P. inundata*	RAM_NA1	*P. ramorum* NA1
BIL	*P. bilorbang*	IPO	*P. ipomoeae*	ROS	*P. rosacearum*
BIS	*P. bisheria*	IRA	*P. iranica*	RUB	*P. rubi*
BOE	*P. boehmeriae*	KEL	*P.* sp*. kelmania*	SIS	*P. siskiyouensis*
BOT	*P. botryosa*	KER	*P. kernoviae*	SOJ	*P. sojae*
BRA	*P. brassicae*	LAC	*P. lacustris*	SYR	*P. syringae*
CAC	*P. cactorum*	LAT	*P. lateralis*	TEN	*P. tentaculata*
CAP	*P. capsici*	MEA	*P. meadii*	TER	*P. terminalis*
CAS	*P. castaneae*	MED	*P. medicaginis*	THE	*P. thermophila*
CHL	*P. chlamydospora*	MEG	*P. megasperma*	TRI	*P. trifolii*
CIN	*P. cinnamomi*	MEK	*P. megakarya*	TRO	*P. tropicalis*
CIP	*P. citrophthora*	MEL	*P. melonis*	ULI	*P. uliginosa*
CIT	*P. citricola*	MIR	*P. mirabilis*	UNI	*P. uniformis*
CLA	*P. clandestina*	MOR	*P. morindae*	VIG	*P. vignae*
COL	*P. colocasiae*	MUV	*P. multivora*	WAL	*P.* taxon Walnut
CPT	*P. captiosa*	MVC	*P. multivesiculata*	×ALN	*P.* ×*alni*
CRA	*P. crassamura*	NEM	*P. nemorosa*	×ATT	*P.* ×*attenuata*
CRY	*P. cryptogea*	NIC	*P. nicotianae*	×CAM	*P.* ×*cambivora*
CRY_B	*P. cryptogea f.sp. begoniae*	NIE	*P. niederhauseri*	×CIP	*P.* ×*citrophthora*
DRE	*P. drechsleri*	NOV	*P.* sp. *novaeguineae*	×HEN	*P.* sp. ×Hennops
ERY	*P. erythroseptica*	OCC	*P. occultans*	×HET	*P.* ×*heterohybrida*
EUR	*P. europaea*	PAL	*P. palmivora*	×INC	*P.* ×*incrassata*
FAL	*P. fallax*	PAS	*P. parvispora*	×LAC	*P.* ×*lacustris*
FRA	*P. fragariae*	PER4	*P.* sp. Peru4	×MUF	*P.* ×*multiformis*
GAL	*P. gallica*	PIF	*P. pinifolia*	×PCR	*P.* ×*pseudocryptogea*
GEM	*P. gemini*	PIN	*P. pini*	×PEL	*P.* ×*pelgrandis*
GLO	*P. glovera*	PIS	*P. pistaciae*	×SER	*P.* ×*serendipita*
GON	*P. gonapodyides*	PLU	*P. pluvialis*	×STA	*P*. ×*stagnum*
GRE	*P. gregata*	PLV	*P. plurivora*	HpAVE	*Halophytophthora avicenniae*
HED	*P. hedraiandra*	POL	*P. polonica*	HpPOL	*Halophytophthora polymorphica*
HEV	*P. heveae*	POR	*P. porri*	PpCIT	*Phytopythium citrinum*
HIB	*P. hibernalis*	PRI	*P. primulae*	PpVEX	*Phytopythium vexans*
HUM	*P. humicola*	PSR	*P. pseudosyringae*		
HUN	*P.* sp. *hungarica*	PST	*P. pseudotsugae*		

### Flow cytometry

Nuclei were prepared and stained using the CyStain PI Absolute P kit (Sysmex Partec, Görlitz, Germany) and flow cytometry was carried out using a Partec PAS III flow cytometer (Sysmex Partec, Görlitz, Germany). Details of the methods are described in detail in Jung et al. (2017c), except that in most cases isolates were measured in three biological replicates, each time in three technical replicates (nine measurements in total). Each biological replicate was conducted using a different preparation of nuclei, starting from a separate subculture, on a different day. The technical replicates were separate measurements on the same preparation of nuclei, within the same day. The coefficient of variation (CV) was calculated based on the three biological replicates (*n* = 3). Sharper peaks and lower CVs between measurements were obtained when cultures were grown in 2% V8 broth compared to 5% or higher (data not shown). Histograms were obtained on a linear scale and no gating was applied. For some *Phytophthora* isolates with a smaller genome we used *Arabidopsis thaliana* Col-0 petals (2C = 0.32 pg = 314 megabase pairs (Mbp); Bennett 2003) as a reference instead of leaves of *Raphanus sativus* cv. Saxa (2C = 1.11 pg = 1086 Mbp; Doležel et al. 1992). The genome size was expressed in Mbp/2C or pg/2C (expression per 2C is regardless of the ploidy level of the genome). Conversion from pg to Mbp was made by multiplying the DNA content in pg by 978 Mbp/pg/2C (Doležel and Greilhuber 2010).

### Genotyping-by-sequencing

#### Library preparation and sequencing

GBS libraries were prepared from the isolates listed in Table S1 according to the method described by Poland et al. (2012), with some modifications. Approximately 100 ng DNA of each sample was digested for 15 min at 37 °C with 1 μl *Pst*I FastDigest, 1 μl *Hpa*II FastDigest and 2 μl FastDigest buffer (Thermo Fisher Scientific) in a total volume of 20 μL. The *Pst*I adapter containing the barcodes (0.02 μM) and the *Hpa*II Y-shaped adapter (0.3 μM) were ligated to the restriction sites (2 h at 22 °C) using 200 units T4 ligase and T4 DNA ligase buffer (NEB), followed by enzyme inactivation at 65 °C during 20 min. The ligate was cleaned up using Sera-Mag SpeedBeads (Thermo Fisher Scientific) and DNA was eluted in 30 μl 0.1x TE. In total, 10 μl DNA was used in a PCR mix containing Taq 2x Master Mix (NEB) and 0.75 μM each of primer IlluminaF_PE and IlluminaR_PE in a total volume of 50 μL. The fragments were amplified in a PCR-machine (Applied Biosystems 9800 Fast Thermocycler) using a 30 s denaturation step at 95 °C followed by 12 cycles of 30 s at 95 °C, 20 s at 65 °C, and 30 s at 68 °C. Subsequently, 25 μl of each sample were cleaned using Sera-Mag SpeedBeads and eluted in 30 μl 0.1x TE. Library quality was assessed by quantifying the DNA with a Quantus Fluorometer and by visualizing the fragment length profile using a QIAxcel Advanced System (Qiagen). The amount of library DNA of each sample was adjusted based on the genome size of the species (or a closely related species if the genome size was not known), in order to sequence a similar number of genome equivalents and thus a more constant sequencing depth per locus. Mean locus depths per sample were calculated and visualized as a histogram (Figure S1). In total, 96 samples with 96 unique barcodes were pooled for one lane of Illumina HiSeq3000 sequencing. This number was based on saturation analyses (number of unique GBS loci identified at increasing total number of sequence reads mapped per library; see below for detailed analyses) to avoid insufficient read depths. The libraries were paired-end sequenced on an Illumina HiSeq3000 instrument (2 × 150 bp) at the Oklahoma Medical Research Foundation Genomics Facility (Oklahoma City, OK, USA).

#### GBS data analysis

##### Data assembly

The sequence reads were submitted to a custom pre-processing pipeline which consisted of: (1) demultiplexing and sorting of the reads based on the barcodes using the GBSX software v1.1.5 (Herten et al. 2015); (2) trimming of the adapters, removal of the remainder of the restriction sites at the 5′- and 3′-ends and discarding reads smaller than 10 bp using cutadapt v1.16 (Martin 2011) and FastX toolkit v0.0.14 (https://hannonlab.cshl.edu/fastx_toolkit/index.html); (3) merging of the forward and reverse reads that had a minimum overlap of 10 bp using PEAR v0.9.8 (Zhang et al. 2014); and (4) several filtering steps using FastX toolkit, prinseq-lite (Schmieder and Edwards 2011), OBITOOLS v1.2.5 (Boyer et al. 2016) and pairfq 0.14 (https://github.com/sestaton/Pairfq). In this last step reads with a mean phred quality score of less than 25, reads that were shorter than 30 bp or those that contained any Ns were discarded. Additionally, singletons, non-merged paired reads and merged reads that still contained a *Pst*I or *Hpa*II restriction site were discarded using the “obigrep” function of OBITOOLS. The trimming and quality filtering script were parallelized using “GNU parallel” (Tange 2018).

##### Loci identification

We performed GBS on hundreds of *Phytophthora* isolates but the reads were not mapped to a particular reference genome sequence, nor were reference genome sequences used for SNP discovery. There were two reasons for this: (1) reference genomes were available for only 21 *Phytophthora* species at the time of the analysis; and (2) analysis of the species themselves was not in the scope of this study. Instead, we used a reference-free approach as implemented in GibPSs (Hapke and Thiele 2016) to construct a tag collection covering all possible genome complements in the dataset including genome sequences not covered by the 21 sequenced genomes. All merged reads were consecutively analysed with *indloc*, *poploc,* and *indpoploc* to identify loci and additionally to correct errors by *indloc*. This was done using the programs’ default settings, except for the frequency threshold method in *indloc* where we used a character frequency threshold of 0.1 (instead of 0.2). These settings were chosen after testing different parameter combinations and checking reproducibility of biological and technical replicates (see below for detailed analyses). Loci with a length of 32 to 250 bp were selected with *data selector*. *Indelchecker* was used to identify loci that contained indels and these loci were removed from the dataset. *Depth analyzer* was used to assess the read depth of the loci and loci that were sequenced extremely deeply were subsequently discarded, as these could belong to repeats, transposons, mitochondria, etc. This was done by removing loci with a median depth percentage of less than 1% and a median scaled depth of more than 0.1. Finally, split loci were removed from the dataset. Isolates with a deviating curve in the depth analysis graph were potentially contaminated with non-*Phytophthora* loci and were thus omitted from the analysis. Additionally, all remaining loci in the dataset were used in a local BLAST search against a custom database composed of a representative set of prokaryotes (“ref_prok_rep_genomes” from ftp://ftp.ncbi.nlm.nih.gov/blast/db), refseq genomes of fungi (“fungi_XX_genomic” from ftp://ftp.ncbi.nlm.nih.gov/refseq/release/fungi), the human genome (build 38) and all available whole-genome shotgun sequence data of the genus *Phytophthora* in the NCBI WGS database. Isolates for which more than 250 GBS tags had significant BLASTn hits (E < 1e-4) with non-*Phytophthora* sequences were removed from the dataset and the GibPSs pipeline was restarted, as loci derived from contaminations could lead to inflated numbers of GBS loci for that isolate.

##### Determination of shared loci and allele similarity

The GibPSs output table containing the identified genotypes (genotypes.txt) was exported and used as input for a custom-made Python script that performs a pairwise comparison of all isolates and determines: (1) how many loci an isolate contains; (2) how many loci are shared for each pair; and (3) the allele similarity of these shared loci based on the haplotype data, i.e. the percentage of the loci that have at least one identical allele in common (see Fig. 1c). For each pair of isolates the Dice similarity coefficient (Dice 1945) was calculated using the number of loci of each isolate and the number of loci they share. This coefficient was subsequently multiplied with the allele similarity, resulting in a combined similarity index (CSI), expressed as a value between 0% (no alleles shared) and 100% (all loci shared and allele similarity of 100%). This index was used for species delimitation of non-hybrid isolates. Saturation curves were constructed showing the dependency of “estimated genetic similarity scores” (shared loci and % allele similarity) versus “total number of reads per sample” (Figure S2). The curves were calculated based on a subset of the reads (computationally subsampled as a random set of all reads per sample at 0.25, 0.5, 0.75, 1, 1.5, 2, 3, and 3.5 M) compared to all available data per sample as reference for a selected number of isolates. This was done either within a single sample (Figure S2a-b; i.e. comparing subsampled datasets to all available reads of that sample), or between samples (Figure S2c-f; i.e. comparing subsampled datasets of the first sample against all available reads from the second sample. The number of shared alleles, number of unique alleles and allele similarity was also calculated for biological and technical replicates in order to evaluate the reproducibility of the technique (Table S3).

##### Allele count analysis

The GibPSs output table containing the identified genotypes (genotypes.txt) was read into Rstudio v1.0.153 running R v3.4.3 and stored as a data table, a high performance version of a data frame, using the data.table package v1.11.2 (Dowle and Srinivasan 2018). For each isolate the number of loci with one, two, three and four alleles was calculated. These data were visualized as a stacked bar chart using ggplot2 v2.2.1 (Wickham 2016).

### Hierarchical clustering and Phylogenomic analyses

GBS resulted in two types of data. Absence/presence of GBS loci was used in hierarchical clustering while SNP data was used for phylogenomic analyses. Hybrids and suspected hybrids were removed from the GBS dataset and only the data of representative genotypes (max. three per species) from the non-hybrid species (and species that are suspected to be non-hybrids) were retained.

For the hierarchical clustering, the locus data from a selection of non-hybrid isolates (144 isolates; not more than three genotypes per species) was read into Rstudio and converted to binary data (presence = 1, absence = 0). Subsequently a dendrogram was inferred using the supraHex package v1.26.0 (Fang and Gough 2014) with the Euclidean distance, Minimum Evolution algorithm with ordinary least-squares fitting (“fastme.ols”) and 500 bootstrap replications.

For the phylogenomic analysis we applied two phylogenetic methods on the GBS data: (1) a method based on the concatenated SNPs as identified by GibPSs; and (2) a coalescent-based method (ASTRAL-III). For both methods, a dataset was made that consisted of one genotype per non-hybrid species (97 isolates). This reduced dataset was used to select the loci that were present in at least 80% or 30% of these isolates (referred to below as loci80 and loci30, respectively) using a custom R script. This corresponded to 29 and 1610 loci, respectively. Phylogenetic analyses were subsequently applied on the selection of non-hybrid isolates (144 samples, see hierarchical clustering). For the concatenation-based method, all loci were concatenated and aligned into a single fasta file using a custom Perl script. Next, the fasta file was converted to a phylip file using jModeltest2 v2.1.10 (Darriba et al. 2012) and all invariant SNPs were removed from the alignment using the “ascbias.py” script from https://github.com/btmartin721/raxml_ascbias. This resulted in 1062 and 61,111 variant SNPs for loci80 and loci30, respectively. Phylogeny was assessed using maximum likelihood with the program RAxML v8.2.10 (Stamatakis 2014), using the GTRCAT model without rate heterogeneity with a correction for ascertainment bias. No invariant SNPs appeared in the dataset, thus the Lewis correction was used. Statistical support was calculated by applying 500 bootstrap runs.

For the coalescent-based method, the sequence of each locus was aligned using a custom Perl script resulting in *per locus* alignment files. The fasta files were converted into phylip files and invariant SNPs were removed as described above. A tree was inferred for each locus using RAxML, with the same settings as with the concatenation-based method but without bootstrapping. We then applied ASTRAL-III v5.6.2 (Zhang et al. 2018) to the resulting maximum likelihood trees. Local posterior probabilities (Sayyari and Mirarab 2016) were used for assessing the branch support. All trees were visualized using FigTree v1.4.4 (Rambaut 2018).

## RESULTS

### Flow cytometry

Flow cytometry analyses (Table S2) revealed highly variable genome sizes among the *Phytophthora* isolates, ranging from 112 ± 11 Mbp/2C to 844 ± 32 Mbp/2C, with a median of 187 Mbp/2C. Approximately 63% of the species had a genome size between 120 and 200 Mbp/2c. The average CV across all samples was 3.1%.

The hybrids *P*. ×*serendipita* (a hybrid of the diploid species *P. cactorum* and *P. hedraiandra*) and most isolates of *P*. ×*pelgrandis* (a hybrid of *P. cactorum* and *P. nicotianae*) had a genome size of 182 ± 5 Mbp/2C and 184 ± 4 Mbp/2C, respectively. This was similar to the genome size of the parental species *P. cactorum* (182 ± 5 Mbp/2C), *P. hedraiandra* (188 ± 2 Mbp/2C) and *P. nicotianae* (184 ± 6 Mbp/2C). *P*. ×*pelgrandis* isolate 15/007 had an exceptionally small genome size (119 Mbp/2C), which is smaller than the genome sizes found in both parents.

A very large genome size (> 300 Mbp/2C) was recorded for all clade 7 species, with the exception of *P. fragariae¸ P. europaea,* and *P. parvispora*. For *P.* ×*alni*, *P. ×multiformis* and *P. ×incrassata* this even exceeded 500 Mbp/2C. Given the average genome size of the diploid *P. uniformis* (330 Mbp/2C) and tetraploid *P. ×multiformis* (653 Mbp/2C), the monoploid genome size is expected to be 165 and 163 Mbp/1C, respectively. All *P.* ×*alni* isolates, except isolate AC03, had a genome size that corresponds to a triploid state, confirming the findings of Husson et al. (2015), while the genome size of *P.* ×*alni* isolate AC03 (844 Mbp/2C) suggests a pentaploid (or a degenerate hybrid with a higher ploidy level). However, chromosome counts are needed to decisively determine the ploidy level of this isolate. We also recorded a large genome size (> 300 Mbp/2C) for several other species (e.g. *P. cinnamomi, P. niederhauseri*). The smallest genome sizes (between 110 and 140 Mbp/2C) were mostly recorded for species from clade 2 and clade 9, but also *P. heveae* (clade 5), *P*. taxon Walnut (clade 6a), *P. ramorum* (clade 8c), and *P. kernoviae* (clade 10), have a genome size in this range.

### Genotyping-by-sequencing and species identification

On average we obtained 3.8 M read pairs per isolate, ranging from 726,474 for *P. quercetorum* isolate TJ025 to 12,890,320 for *P.* ×*alni* isolate AC03. Overall, we could identify 1,762,508 loci that harbour 4,857,367 SNPs in a total of 661 samples (incl. replicates) that represent 132 hybrid and non-hybrid *Phytophthora*, two *Halophytophthora,* and two *Phytopythium* taxa. The average read depth per locus per isolate was 71, with 96.6% of the loci having an average read depth of at least 15 and 91.2% of at least 20 (Figure S1). To obtain accurate genotype calling, a minimum of 8 reads per locus, and 20 reads per locus yield a 99% chance to detect all alleles at least once in diploids and tetraploids, respectively (Griffin et al. 2011; Joly et al. 2006). The average locus length was 126.6 bp and the median 120.0 bp (range 32–250 bp). Taken together, this means that about 1 in 43 nucleotides carry a polymorphism across the complete dataset spanning 661 isolates belonging to > 100 different species. There were no loci common to all isolates, underlining the high genome diversity within the genus and the need for reference-free data analysis. Saturation curves in a selection of representative isolates were used to investigate the relationship between total read depth and accuracy of our genetic similarity scores (the number of shared loci and the allele similarity). We analysed the data within samples (Figure S2a-b) and between samples of diploid (Figure S2c-d) and polyploid (Figure S2e-f) hybrids. For diploid non-hybrid species (e.g. *P. cactorum, P. hedraiandra*) 1 M reads per sample were sufficient, while for diploid hybrids (e.g. *P.* ×*serendipita, P.* × *pelgrandis*) about 2 M reads were sufficient to reach a plateau and a higher total number of reads did not further increase the number of loci detected per sample (Figure S2a and S2c). For polyploid hybrids with a more complex genome such as *P.* ×*heterohybrida* and *P.* ×*incrassata* 3 M to 4 M reads were necessary to reach complete saturation (Figure S2a and S2e). In all cases, allele similarity stabilizes with at least 1.5 M reads per sample (Figure S2b, d, f). Replicate samples were analysed to assess the reproducibility of GBS. In some cases, we detected a number of alleles that were not shared between replicates, but the allele similarity between replicates was always higher than 99.89%, therefore we preferred to use this measure to compare alleles between samples (Table S3). The cumulative sequence length of all GBS loci per species was calculated for *P. cactorum*, *P.* ×*alni,* and *P. kernoviae* and corresponds to 4.8%, 4.6%, and 5.2%, of the total genome size in these species, respectively.

**Fig. 2:**
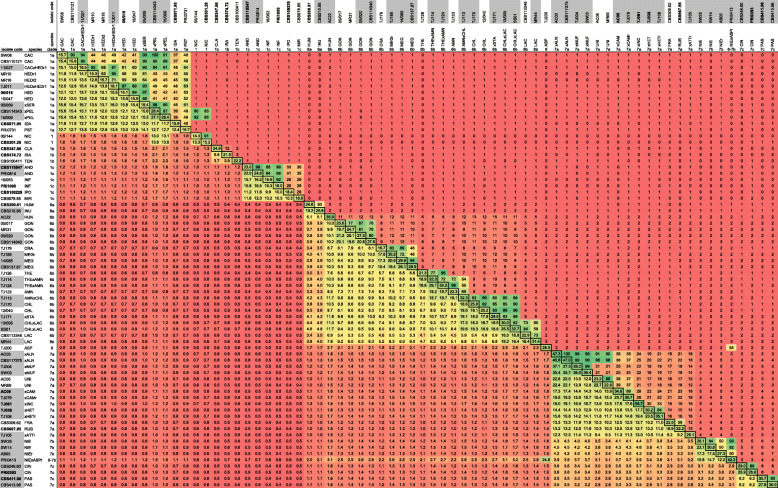
Pairwise comparison of GBS loci of a selection of isolates from *Phytophthora* clades 1, 6 and 7. Isolate codes in bold are ex-type strains or authentic strains (see Supplementary Table 1 for details). Isolate codes marked in grey have been identified as hybrids, either previously or in this study. Numbers in the diagonal (with border) indicate the number of loci of each isolate. Numbers under the diagonal represent the number of loci shared among each pair of isolates (expressed as thousands). The colour code in this part of the table denotes the number of shared loci and has a range from green (highest number = 47,273 loci for *P. ×alni* isolate AC03), to yellow (5000 shared loci) and to red (lowest number = 357 loci shared between *P. humicola* isolate CBS200.81 and *P. ipomoeae* isolate CBS109229). Numbers above the diagonal represent the allele similarity (percentage of the shared loci in which at least one allele is identical) of the shared loci (expressed as a percentage). The colour code in this part of the table ranges from green (100%), to yellow (50%) and red (0%). Table S4 contains these data for all isolates in this study

Speciation is associated with accumulated genetic diversity (Fig. 1a), which results in species-specific GBS fingerprints (Fig. 1b). The fingerprints are compared between all samples to identify shared loci and to assess the allele similarity (i.e. the percentage of the shared loci with at least one identical allele). This comparison is displayed in a colour-coded matrix (Fig. 1c, Fig. 2, Table S4) and shows the number of loci per isolate on the diagonal (*cfr*. Figure 1c). This number of loci per isolate varied strongly and ranged from 10,923 loci for *P. occultans* isolate 13/037 to 54,693 loci for *P.* ×*incrassata* isolate TJ061, with an average of 22,958 loci (median = 21,707; SD = 8270; *n* = 661). Table S4 also shows the allele similarity. Many loci were shared between closely related species and the allele similarity of these loci was high (*cfr*. Figure 1b and c). For more distantly related species the number of shared loci and the allele similarity dramatically dropped. For instance, the two closely related subclade 1a species *P. cactorum* and *P. hedraiandra* contained on average 15,565 and 15,914 loci, respectively, of which 11,858 loci (76.2% of the *P. cactorum* and 74.5% of the *P. hedraiandra* loci) were shared between the two species. These shared loci displayed an allele similarity of 42.5%. Subclade 1b species *P. tentaculata* contained on average 21,829 loci of which 1559 loci (7.1%) were shared with *P. cactorum* and displayed an allele similarity of 0.5%. Clade 2a species *P. botryosa* contained 12,136 loci and had only 462 shared loci (3.8%) with *P. cactorum* (allele similarity of 0.2%)*.* This is reflected in CSI values that decreased from 32.0% for *P. cactorum* and *P. hedraiandra* (both clade 1a) to 0.05% for *P. cactorum* and *P. tentaculata* (clade 1b) and 0.01% for *P. cactorum* and *P. botryosa* (clade 2a) (Table S5). Within most species, the CSI between different genotypes was higher than 80%. The non-hybrid species *P. meadii*, *P. capsici*, *P. multivora*, *P. pseudosyringae*, *P. palmivora*, *P. gonapodyides*, *P. lacustris*, *P. bilorbang*, *P. ramorum,* and *P. syringae,* contained diverse genotypes with CSI values down to 54.3% (between *P. bilorbang* isolates TJ166 and TJ167). The highest CSI between isolates of distinct species was 54.4%, noted between *P. acerina* isolate 14/013 and *P. plurivora* isolate SS06. Based on these observations we consider isolates with a CSI > 55% as conspecific, while values between 50 and 55% suggest diverging or recently diverged species.

Global analysis of the number of shared loci, the allele similarity and/or the CSI across all isolates revealed that out of the 614 *Phytophthora* isolates, 73 isolates representing 40 species had been incorrectly identified prior to this study. In these cases, species identity was corrected. Some of these corrections involve putative novel species, e.g. *P. hedraiandra*-related1, *P. hedraiandra*-related2 and *P. citrophthora*-related. Initial and corrected species names are listed in Table S1.

### Identification and characterization of hybrids

Due to the vast diversity among the species and hybrids in the genus *Phytophthora* and the relatively small number of shared loci between species that are distantly related, traditional methods for hybrid detection proved to be challenging. Initial attempts using likelihood-based ancestry proportion estimation methods (e.g. Admixture; Alexander and Lange 2011) and phylogenetic network methods (e.g. Phylonet; Than et al. 2008) either gave inconsistent or extremely complex results, or were simply not able to capture all introgression events between the individuals, even when testing different population sizes (K) and/or limiting the number of individuals in the analysis (data not shown). Therefore, we developed a novel strategy to easily detect hybrids.

This strategy is illustrated in Fig. 1 and comprised a combination of three distinct features typical for hybrid species. First, a hybrid shares GBS loci with more than one species and the loci shared with the progenitors have a high allele similarity (in general more than 70%). This was visualized as a tripartite relationship between hybrid and progenitors in the pairwise comparison of the loci in all samples (Fig. 1c, Fig. 2, Table S4).

Second, a hybrid always has a larger number of loci compared to the number of loci of its progenitors as it contains the two parental genomes. The level of this increase is directly proportional to the genetic divergence of the parental species because the number of loci of a hybrid is the sum of the shared loci of the parental species plus the unique loci of each parent (see Fig. 1d). Highly divergent progenitors (e.g. species B and C in Fig. 1b) share only a limited number of loci and thus have a larger number of unique loci compared to progenitors that are closely related (e.g. species A and B in Fig. 1b), causing a substantial increase in the number of loci in the hybrid. This feature allowed us to identify hybrids in cases where only one or no progenitor was present in our set of reference isolates, combined with the third feature if necessary.

**Fig. 3:**
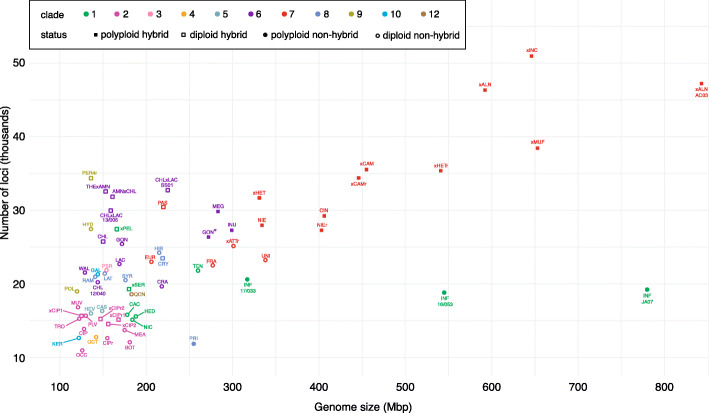
Average number of loci and genome size of *Phytophthora* species. The colour refers to the clade of the species while the shape refers to the hybrid status of the species. Isolates that deviate significantly from other isolates of their species in terms of average number of loci and/or genome size are shown separately, together with their isolate code. The second *P. gonapodyides* point (marked with an asterisk) refers to isolates 05/025, 06/001, 07/004 and 07/007. For isolates with multiple nuclear genome sizes we used the one corresponding to the predominant flow cytometry fluorescence peak (for additional peaks, see Table S2)

The third feature consisted of a combination of a large number of multi-allelic loci (empirically estimated threshold at more than 5% of the loci; see Figure S3a-g) and a large genome size (> 250 Mbp/2C; see Fig. 3). This feature was mainly used for the identification of polyploid hybrids. Loci shared between the parental species may contain SNPs up to a level where they will be classified as two or more alleles (if the level of SNPs becomes too high the two alleles will be classified as two separate loci in the reference-free loci identification method implemented in GibPSs). The allele counts are visualized in a barplot diagram (Fig. 1b, Fig. S3). These hybrids also have a large genome size due to the genome doubling that usually occurs after hybridization between two highly divergent species. However, a large genome size alone does not suffice to discern between diploid and polyploid hybrids as enlarged chromosomes can also generate large genome sizes. Thus, this characteristic should always be evaluated together with the proportion of multi-allelic loci in the barplot.

In clade 1a we identified four hybrid species of which two were previously known, i.e. *P*. ×*serendipita* (*P. cactorum* × *P. hedraiandra*) and *P*. ×*pelgrandis* (*P. cactorum* × *P. nicotianae*). The other two hybrid species are the result of hybridization of a species we designated as *P*. *hedraiandra*-related1 (based on their position in the phylogenetic tree; see below) with either *P*. *cactorum* or *P. hedraiandra*. The observed number of loci in the clade 1a hybrids corresponded well to the expected number as calculated based on the unique and shared loci of the respective parental species (Table 2, e.g. *P. ×serendipita* in Fig. 1d). Also *P. andina,* a known hybrid from clade 1c with *P. infestans* as one of its parental species, shared a large number of loci with *P. infestans* and as expected had more loci (average 23,808) than *P. infestans* (average 19,287).

**Table 2:** Comparison of expected and observed numbers of GBS loci of selected hybrid *Phytophthora* species in clades 1, 6 and 7

Clade	Parental species	Number of parental loci			Hybrid species	Number of hybrid loci		
		Observed	Unique^a^	Shared		Expected^b^	Observed	Shared with parental species
1a	*P. cactorum*	15,565	3707	11,858	*P.* ×*serendipita*	19,621	19,181	15,231
	*P. hedraiandra*	15,914	4056					15,444
1a	*P. cactorum*	15,565	3827	11,738	*P. cactorum* × *P. hedraiandra*-related1	19,243	18,325	14,812
	*P. hedraiandra*-related1	15,416	3678					14,639
1a	*P. hedraiandra*	15,914	3055	12,859	*P. hedraiandra* × *P. hedraiandra*-related1	18,471	18,081	15,363
	*P. hedraiandra*-related1	15,416	2557					14,961
1a	*P. cactorum*	15,565	14,000	1565	*P.* ×*pelgrandis*	28,992	27,550	14,706
1	*P. nicotianae*	14,992	13,427					13,194
6b	*P. thermophila*	21,405	13,904	7501	*P. thermophila* × *P. amnicola*	36,190	32,562	19,368
	*P. amnicola*	22,286	14,785					19,083
6b	*P. amnicola*	22,286	14,828	7458	*P. amnicola* × *P. chlamydospora*	35,077	32,010	17,966
	*P. chlamydospora*	20,249	12,791					19,378
6b	*P. chlamydospora*	20,249	15,356	4893	*P. chlamydospora* × *P. lacustris*	38,305	31,337	17,760
	*P. lacustris*	22,949	18,056					16,028
7a	*P. uniformis*	22,757	10,059	12,698	*P.* ×*alni*	46,703	45,128	21,957
	*P.* ×*multiformis*	36,644	23,946					34,259
7b	*P. niederhauseri*^c^	15770^c^	14,183	1587	*P. niederhauseri* × *P. asparagi*-related	42,486	42,192	15,770
6	*P. asparagi*^d^	28,303	26,716					24,851

^a^Number of unique loci calculated as the average number of loci for the species minus the average number of loci shared with the other parental species

^b^Expected loci in the hybrid calculated as the sum of the number of unique loci for each parental species and the number of loci shared between the parental species

^c^Because *P. niederhauseri* is a hybrid itself, the theoretical number of loci for non-hybrid *P. niederhauseri* was estimated based on the number of shared loci of the *P. niederhauseri* × *P. asparagi*-related hybrid and the hybrid *P. niederhauseri* isolates that have an allele similarity = 93%

^d^*P. asparagi*-related is not present in our set of isolates so *P. asparagi* was used instead, assuming it has a similar number of loci

In some cases, not only the hybrids but also their parental genotypes could be identified based on the allele similarity between the hybrid and its progenitors. For instance, all *P. ×serendipita* and nearly all *P. ×pelgrandis* isolates have a *P. cactorum* genotype that is either identical or very closely related to that of isolates SW08 and 07/008. The parental *P. hedraiandra* genotype of the *P. ×serendipita* hybrids is closely related to that of ex-type isolate 06/018 (= CBS111725). In contrast, the parental *P. nicotianae* genotype of the *P. ×pelgrandis* isolates is not present in our set of isolates.

In clade 2, isolate CBS111726, originally identified as *P. citrophthora*, appeared to be a novel hybrid of *P. occultans* and a species closely related to *P. citrophthora*, as it shared nearly all *P. occultans* loci (with 98.8% allele similarity with most *P. occultans* isolates) and 10,705 of the 13,544 *P. citrophthora* loci (with 77.9% allele similarity). Based on the ITS and *cox*I sequencing *Phytophthora* clade 2 isolates TJ100, TJ101, TJ103 and TJ104 were previously designated as hybrids (Jung et al. 2017a). We therefore conclude that TJ093, TJ102 and TJ184 are also hybrids. The parental species of isolates TJ093, TJ100, TJ101, TJ102, and TJ103 are not in our dataset. One of the parental species of isolates TJ104 and TJ184 is closely related to *P. citrophthora* (10,223 and 10,395 loci in common with an allele similarity of 69.8 and 79.0%, respectively). TJ104 also has 13,254 loci in common (with 95.4% allele similarity) with isolates TJ100, TJ101, TJ102 and TJ103, indicating that all of these isolates share one as yet unknown parental species.

**Fig. 4:**
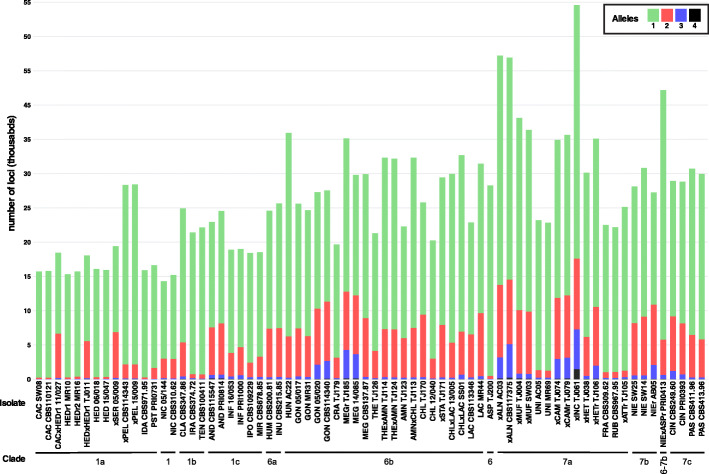
Number of GBS loci with one to four alleles in representative *Phytophthora* isolates of clades 1, 6 and 7. The data for isolates of all clades are presented in Figure S3a-g

In clade 6b, isolates TJ114, TJ115, TJ116, TJ117, TJ121, TJ122, TJ124 and TJ125 are hybrids of *P. amnicola* and *P. thermophila* (or a species closely related to *P. thermophila*). The *P. thermophila* parent of isolate TJ124 is genetically almost identical to *P. thermophila* isolates TJ127 and TJ165 (99.0% allele similarity). Based on their large number of loci, all isolates of *P. chlamydospora* were identified as hybrids, except for isolate 12/040. For *P. gonapodyides* we found two groups. One group had an average of 24,855 loci and an average genome size of 172 Mbp/2C. The second group contained 27,119 loci on average and had an average genome size of 271 Mbp/2C. This second group also had a large number of triallelic loci (≥ 5%; Fig. 4) indicating they are likely polyploid hybrids. For *P. lacustris* we found one isolate (MR44) with a very large number of loci (31,448 loci) which is probably the result of hybridization with an unknown species. The expected number of loci of the hybrids *P. thermophila* × *P. amnicola*, *P. amnicola* × *P. chlamydospora,* and *P. chlamydospora* × *P. lacustris* were higher than those observed (Table 2). Especially for *P. chlamydospora* × *P. lacustris* the difference was considerable, primarily due to the relatively limited presence of *P. lacustris* loci in the hybrids (only 16,028 loci out of the 22,949 loci). The high diversity of *P. lacustris* could be the reason for this discrepancy.

In clade 7a, *P*. ×*alni* has the expected combination of the loci of progenitors *P. uniformis* and *P*. ×*multiformis* (Table 2). The loci that are shared between the *P*. ×*alni* isolates and the isolates of *P. uniformis* have an average allele similarity of 97.2%, except for *P*. ×*alni* isolate AC03, which has an allele similarity of only 90.5%. Genome sizes of *P*. ×*alni* (± 505–565 Mbp/2C) are intermediate to *P. uniformis* (± 348 Mbp/2C) and *P*. ×*multiformis* (± 653 Mbp/2C), except for *P*. ×*alni* isolate AC03 which has a much larger genome size of 844 Mbp/2C. The parental species of *P*. ×*multiformis* and the other known hybrids in clade 7a, *P*. ×*cambivora*, *P*. ×*cambivora*-related, *P*. ×*heterohybrida,* and *P*. ×*incrassata*, have not been described previously and were also not detected in our set of isolates. *Phytophthora* ×*cambivora*, *P*. ×*cambivora*-related and four isolates related to *P*. ×*heterohybrida* (TJ106-TJ109) had a large number of triallelic loci, while *P*. ×*incrassata* had a relatively large number of loci with four alleles, indicating that all these species are polyploid hybrids.

In clade 7b, the *P. niederhauseri* isolates contained a large number of loci, indicating the hybrid nature of this species. Two genotypes could be discerned based on their number of loci (27,935 vs 30,688) and their allele similarity (83.9%). Interestingly, isolate PRI0413, originally designated as *P. asparagi,* contained 42,180 loci. Of those loci, 15,826 loci are shared with *P. niederhauseri* genotype 1, 14,720 loci with *P. niederhauseri* genotype 2, and 24,812 loci with *P. asparagi* (from clade 6). These data indicate that this isolate is a hybrid between species from clades 6 and 7, and is thus the first reported natural interclade hybrid. The allele similarity was 93.3% with the *P. niederhauseri* isolates of genotype 1 and 82.0% with those of genotype 2. The allele similarity with the three *P. asparagi* isolates in our collection was only 53.6%, suggesting the second parent could be a species closely related to *P. asparagi* rather than *P. asparagi* itself.

In clade 8a, the *P.* ×*pseudocryptogea* hybrids, identified based on ITS and *cox*I sequencing (data not shown), have only 17,409 loci. Due to the absence of the parental species in our set of isolates we could not directly confirm their hybrid status. Among the *P. cryptogea* isolates we found two clearly distinct groups. The first group consists of non-hybrids, represented by isolates 05/059, CBS418.71, SW35 (originally misidentified as *P. drechsleri*), *P. cryptogea* ex-type strain CBS113.19 and the *P. erythroseptica* ex-type strain CBS129.23, which possess on average 17,182 loci. The second group contains hybrid species with an average of 23,647 loci. Their parental *P. cryptogea* genotype is in most cases closely related to the genotype of CBS417.71.

In clade 8b, *P. porri* isolate CBS138.87 possesses 17,655 loci, which is notably more than the average of the other *P. porri* isolates and *P. primulae*. This isolate shares on average 8925 loci with *P. porri* and 10,199 loci with *P. primulae.* The number of loci (18,231) approximates the sum of the unique *P. porri* and *P. primulae* loci (6312 and 7017 loci, respectively) and their shared loci (4902 loci). However, the allele similarity is only 62.3% with *P. porri* and 71.1% with *P. primulae*. Hence, isolate CBS138.87 appears to be a hybrid between *P. porri* and *P. primulae*, or at least of very closely related species.

In clade 8d the *P. austrocedrae* isolate included in the analysis contained a relatively large number of loci and large numbers of bi- and triallelic loci (Figure S3f) compared to the other clade 8d species *P. syringae*. This suggests this *P. austrocedrae* isolate could also be a hybrid.

In clade 9a1 both *P.* sp. Peru4-related and *P.* ×Hennops-related were found to be hybrids, based on their large number of loci (on average 39,418 loci) of which 22,709 are shared between the two taxa. These loci are also shared with *P. hydropathica* (22,873 and 22,233 loci shared with *P.* ×Peru4-related and *P.* ×Hennops-related, respectively), indicating that this species is one of the progenitors of these two hybrids. The allele similarity of the shared loci (88%) points to a *P. hydropathica* genotype not present in our set of isolates.

### Discerning diploid and polyploid hybrids

Figure 2 shows the relationship between the number of GBS loci and the genome size as determined by FCM. Polyploid hybrids such as *P.* ×*alni* are located in the upper right corner of the graph, as they combine a large number of loci with a large genome size. Also, these hybrids have a large number of triallelic loci (Fig. 4 and Figure S3a-g).

Diploid hybrids such as *P*. ×*serendipita* and *P.* ×*pelgrandis* are located in the vicinity of their parents in the left part of Fig. 2, as their genomes have a similar size. Their vertical position in the graph depends on the genetic divergence between the parental species.

Not only the previously described polyploid hybrids *P*. ×*alni*, *P*. ×*multiformis*, *P*. ×*cambivora*, *P*. ×*heterohybrida,* and *P*. ×*incrassata* combine a large number of loci with a large genome size, but also species from several other (sub)clades: *P. inundata* (clade 6a), *P. megasperma* and some isolates of *P. gonapodyides* (clade 6b), *P. niederhauseri*, *P. niederhauseri*-related (clade 7b), and *P. cinnamomi* and *P. parvispora* (clade 7c) (Fig. 3). Most of these species possess a relatively large number of triallelic loci, suggesting allopolyploidy. Autopolyploid species such as *P. infestans* have a large genome size but the number of loci does not increase proportionally. *Phytophthora uniformis*, *P. fragariae* and *P. tentaculata* also have a limited number of loci and hardly any triallelic loci (Fig. 4) but a relatively large genome size. These species are positioned in the lower half of the graph (Fig. 3). This could indicate that their genome was at least partially duplicated.

### Phylogenomic analysis

#### Hierarchical clustering

The dendrogram resulting from the minimum evolution analysis of the binary (absence/presence) locus data (Figure S4) showed excellent correspondence with the clades and subclades that were defined in previously published studies (Blair et al. 2008; Cooke et al. 2000; Jung et al. 2017b; Yang et al. 2017). There were two exceptions, however. First, subclade 8b was separated from the other clade 8 subclades and clusters together with clade 10 species *P. boehmeriae*, *P. kernoviae,* and *P. morindae*. In turn, these species are separated from the other clade 10 species, *P. gallica*.

#### Concatenation- and coalescent-based phylogeny

Inferring the phylogenomic tree of 144 selected isolates using the concatenated SNPs from loci30 resulted in a high bootstrap support (> 70) for most branches (Figure S5a). Without exception, the clades and subclades corresponded with those previously published (Blair et al. 2008; Cooke et al. 2000; Jung et al. 2017b; Yang et al. 2017).

The topology of the loci30 tree fitted the data of the shared loci and allele similarity better than the loci80 tree. The latter one displayed a low bootstrap value for many branches, both between and within clades (Figure S5b). Additionally, in the loci80 tree clades 9 and 10 were not resolved, *P. asparagi* did not cluster within clade 6 but was positioned between clades 6 and 8, and clade 8b was placed next to clades 4 and 5.

Similar results were obtained with coalescent-based phylogeny using ASTRAL-III: overall there was stronger statistical support in the tree based on loci30 both within and between clades (Figure S5c). In the ASTRAL tree of loci80, subclades 2d, 2e, 8b, 9a1 and 9a2 had been erroneously separated from their main clade (Figure S5d).

**Fig. 5:**
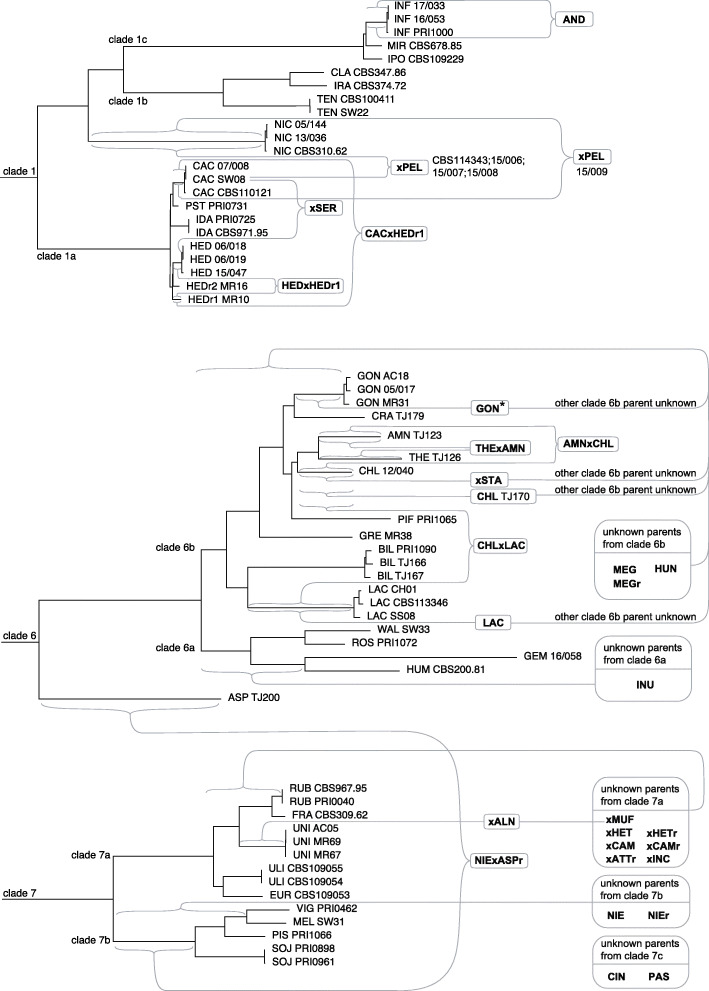
Phylogenomic trees of *Phytophthora* clades 1, 6 and 7 (subtrees excerpted from Figure S6, which contains all species used for the phylogenomic analyses). Hybrids are indicated in bold, positioned separately and linked to their parental species. If a parental species is unknown, the accolade points to the longest branch of the subclade to which the parental species belongs. If the genotype of (one of) the parental species is unknown, accolades are placed at the branch in which the parental species reside. The *P. gonapodyides* hybrids indicated with an asterisk refer to isolates 05/003, 05/020, 05/025, 06/002, 07/004, 07/007, 08/001, CBS114340, MR30, MR37, SS07 and TJ181

The concatenation-based phylogenomic tree of loci30 was used to anchor the hybrids to their progenitors (Fig. 5, Figure S6). When the progenitors were not known, the hybrids were anchored to the branch where their progenitors reside in.

## DISCUSSION

Our objectives were to apply genome-wide fingerprinting (i.e. GBS), supplemented with FCM, to better identify and characterize *Phytophthora* hybrids and to use this information to construct an improved phylogeny of the genus. We present a method that is highly reproducible, and that can be applied to a wide range of other organisms. One critical point is the availability of non-contaminated DNA. It is essential to review the data for the presence of sequence reads from non-target organisms, even when no visible contamination of the cultures is present. Contaminating sequences inflate the number of loci from the target organism, which may erroneously suggest hybrid status, and can hamper correct phylogenetic inference. Performing a BLAST search with the identified *Phytophthora* loci to a custom made database containing sequences of possible fungal and bacterial contaminants was an important step to prevent this artefact.

### Power of GBS for species identification

Genome sequence data from tens of thousands of GBS loci allowed unprecedented high resolution genomic fingerprinting, and were used for sample identification at species level and beyond. It can help to elucidate species complexes in which morphological analysis and even sequence data from barcoding genes provide insufficient information. Here, we introduce a novel similarity index (combined similarity index, CSI) which combines two types of similarity measures, i.e. the number of shared GBS loci between isolates and the SNP-based allele similarity of these loci. This similarity index expresses the genomic divergence between two non-hybrid isolates and was used as an objective measure for species delimitation. Based on our empirical calibration, which was based on sets of species with known related and unrelated identity, we show that CSI values higher than 55% consistently indicate identical species, while CSI values lower than 50% consistently indicate distinct species. In the range between 50 and 55%, the CSI alone is insufficient for conclusive species delimitation. This is mainly the case for species that display extreme levels of intraspecific genetic diversity. In clade 6b for instance, the 27 distinct non-hybrid *P. lacustris* genotypes display CSI values ranging from 95.3 to 54.0% and the CSI of the three distinct *P. bilorbang* genotypes ranges from 60.4 to 54.3%.

Using the CSI we confirmed the findings of Oudemans and Coffey (1991) and Mchau and Coffey (1994) that *P. arecae* isolate CBS148.88 is a genotype of *P. palmivora* (average CSI of 61.9%) and not a separate species. Another example concerns *P. cactorum* and *P. hedraiandra,* closely related species that are difficult to distinguish based on morphology, AFLP and gene sequence data of three loci, on which basis they were previously suggested to be conspecific (Pánek et al. 2016). In contrast, we confirmed their status as separate species based on their low CSI (32.0%). Furthermore, the identity of nine out of 43 clade 8a isolates was corrected. The ex-type strain of *P. erythroseptica* (CBS129.23) for instance, was nearly identical (allele similarity = 99.9%) to the ex-type strain of *P. cryptogea* (CBS113.19) and differed from the other *P. erythroseptica* isolates. This is in agreement with the observations of Robideau et al. (2011) and Q-bank (www.q-bank.eu; Bonants et al. 2013), in which CBS129.23 is the only *P. erythroseptica* isolate with an ITS and *cox*I sequence identical to the *P. cryptogea* ex-type strain. Possibly due to uncertainty about the identity of the ex-type strain, Safaiefarahani et al. (2015) used sequences of *P. erythroseptica* isolates CBS951.87 and CBS956.87 as a reference in their re-evaluation of the *P. cryptogea* species complex. Most probably the *P. erythoseptica* ex-type strain had already been mixed up with *P. cryptogea* immediately after the deposition in the CBS collection (A. De Cock, pers. comm.). Verification of the identity of the strains maintained as *P. erythroseptica* ex-type strains in other culture collections is therefore needed.

In total, our genome fingerprinting method led to correction of species identity in 12% of the samples in our collection, including some from culture collections (Table S1). This high percentage can be partially explained by the preselection of potential hybrids with more complex ITS profiles. On the other hand, misidentifications have already been reported in *Phytophthora* collections (Simamora et al. 2015), as well as in mammalian cell lines (Lorsch et al. 2014) and *Arabidopsis thaliana* accessions in stock centres (Anastasio et al. 2011; Bergelson et al. 2016). Culture collections are extremely valuable for the scientific community and we confirm that validation of accessions via GBS or other techniques (e.g. Pisupati et al. 2017) is appropriate (Bergelson et al. 2016). We also advise researchers to verify the identity of received samples with molecular methods, e.g. by using the two-step approach suggested by Yang and Hong (2018).

### Identification of diploid and polyploid hybrids based on GBS and flow cytometry

We used a strategy for the identification of *Phytophthora* hybrids based on three features that are indicative of hybridization (illustrated in Fig. 1). The first indicator is a large number of GBS loci shared with more than one species and a high allele similarity with the parents (in general more than 70%; see Fig. 2 and Table S4). A second indicator of hybridization is a large number of loci, compared to the parental species, as hybrids combine the shared loci of the two parental species with the unique loci of each of these species (see Table 2 for examples). On average, hybrids indeed contained considerably more loci (28,176 ± 8913, range 13,321 to 50,938) than non-hybrid species (19,112 ± 4723 loci, range 11,002 to 29,091). The third indicator is specific for polyploid hybrids and consists of a combination of a large genome size and a large number of multi-allelic loci. Although little is known about the cellular processes underlying hybridization in *Phytophthora*, it is well established that polyploid hybrids can be formed (Schardl and Craven 2003; Érsek and Nagy 2008). Genome sizes alone cannot be correlated to ploidy levels as different species can have different basic chromosome numbers or chromosome sizes. Counting chromosomes in oomycetes is difficult, however, due to their relatively small sizes (Kamoun 2003; Sansome et al. 1991). Therefore, the genome size should be combined with the data on the proportion of multi-allelic loci.

In total, we identified 43 distinct hybrid taxa in six clades, 24 of which reside in clades 6 and 7, including one interclade hybrid. In clades 1, 2 and 9 we detected only diploid hybrids, while clade 7 contained almost exclusively polyploid hybrids (Fig. 3). Clades 6 and 8 harboured both ex-type strains of hybrids. Although hybridization seems to occur more often than previously recognized, we did not find hybrids in clades 3, 4, 5, 10, and 12. This could in part be explained by a relatively small number of representatives from those clades in our study. Expanding the number of isolates might reveal novel species and/or hybrids. The absence of hybrids could also reflect a reduced hybridization potential of species in these clades because they almost never occur in aquatic settings, which are environments that seem to favour hybridization (Burgess 2015; Jung et al. 2017c, 2018a; Nagel et al. 2013).

Theoretically, the genome size of homoploid hybrids are half the sum of the parental genome sizes. This is indeed the case for *P*. ×*serendipita, P*. ×*pelgrandis,* and *P*. ×*alni*. Hybridization between genetically divergent species can cause sterility. Subsequent polyploidization can overcome this and results in an initial genome size of the sum of the genome sizes of the parental species (Soltis and Soltis 2009). The triploid hybrid *P*. ×*alni* is sterile and cannot survive long in soil or harsh climates without oospores (Redondo et al. 2015). If *P.* ×*alni* were to regain fertility through genome duplication, this could have important epidemiological consequences. Over time, post-hybridization rearrangements of the hybrid genome can, however, significantly influence the genome size. Interspecific hybridization with or without genome doubling and subsequent genome rearrangements can lead to “neospecies” (Chapman and Burke 2007). Especially for plant pathogens one can assume that in polyploids the neofunctionalization of genes can lead to changes in virulence and host range.

Two known hybrids of *P. cactorum*, i.e. *P.* ×*serendipita* and *P.* ×*pelgrandis*, reside in clade 1a. In addition, we identified two novel hybrids, designated *P. cactorum* × *P. hedraiandra*-related1 and *P. hedraiandra* × *P. hedraiandra*-related1. It is striking that all samples isolated from strawberry belong to the same genotype, except for isolate 06/002. Similar results were reported in previous studies (Hantula et al. 1997, 2000). Also, this particular genotype gave rise to, or was at least very closely related to, all *P. cactorum* hybrids included in our study, except for *P.* ×*pelgrandis* isolate 15/009. This indicates that it is more prone to hybridization than the other genotypes and/or that its hybrids have a higher fitness.

No hybrids have previously been formally recognized in clade 2. We identified at least five novel hybrids in clade 2a, three of which involved *P. citrophthora* as a progenitor. One of these, isolate CBS111726, concerns a hybrid with the closely related *P. occultans*. Other related species, such as *P. terminalis* and *P. himalsilva,* were not present in our set of isolates and could also be involved in the other hybrids (Vettraino et al. 2011).

Hybrids among several clade 6b species have previously been described (Burgess 2015; Nagel et al. 2013). Yang et al. (2014) reported *P.* ×*stagnum*, a hybrid between *P. chlamydospora* and a species related to *P. mississippiae*. We also found that clade 6a species *P. inundata* and clade 6b species *P. megasperma*, *P.* sp. hungarica and several *P. chlamydospora* and *P. gonapodyides* isolates are putative hybrids. The high level of sterility among members of clade 6b (Brasier et al. 2003) may in part be explained by the large number of hybrids in this subclade. The tendency of species from this subclade towards hybridization may be facilitated by their riparian natural habitat, where substrates can easily be colonized by more than one species. Several clade 6b species are frequently detected in irrigation water of nurseries, an environment that might promote hybridization. Also, some isolates originating from different countries belong to the same clonal lineage, indicating that plant trade is probably involved in their spread.

Several polyploid hybrids have previously been identified in clade 7a (Brasier et al. 1999; Jung et al. 2017c). It is striking that so many clade 7a hybrid species contain a large proportion of triallelic loci. *Phytophthora* ×*incrassata* even had a significant proportion of loci with four alleles, an indication of allopolyploidy. These observations emphasize the role of hybridization and polyploidization in the evolutionary process of the species in this clade (Jung et al. 2017c). *Phytophthora* ×*multiformis*, one of the progenitors of *P. ×alni*, was itself identified as an allopolyploid hybrid between two species. Consequently, *P.* ×*alni* is a hybrid involving three species (Husson et al. 2015; Ioos et al. 2006), which is reflected in the large number of loci and the large proportion of triallelic loci (Fig. 2, Figure S3e). Similar proportions were found in *P.* ×*cambivora* and *P.* ×*heterohybrida*-related, confirming these species are also hybrids. The large genome size of *P. uniformis* but limited number of loci and small proportion of biallelic loci would indicate autopolyploidization but clearly not hybridization. Brasier et al. (1999) counted 11–13 chromosomes in *P. uniformis,* 10–12 chromosomes in *P.* ×*cambivora* and 10–12 chromosomes in *P. fragariae*. However, they considered *P.* ×*cambivora* and *P. fragariae* as diploid species, which we now know are possible polyploids. Indeed, also *P. fragariae* as well as *P. tentaculata* had genome sizes larger than 250 Mbp/2C but only had a relatively small number of loci and a small fraction of biallelic loci, indicating that these species might also be autopolyploids. More reliable and extensive chromosome counts are however necessary to correctly determine the ploidy level of species in the genus *Phytophthora* and to assess its correlation with the genome size. Due to the speed by which generative cycles occur in *Phytophthora* it is conceivable that processes like whole genome duplication (WGD) and hybridization occur more rapidly than in many other organisms. Especially in the case of pathogens, hybridization followed by WGD might therefore be a driving force in speciation and specialization.

In clade 7b we identified *P. niederhauseri* as a hybrid, based on genome size and the large number of loci. We observed that the species consists of at least two genetically distinct groups. The *P. niederhauseri*-related isolates are presumably hybrids from three parental species, possibly involving *P. niederhauseri* or one of its progenitors. Interestingly, we also identified a hybrid between clade 7b species *P. niederhauseri* and a clade 6 species closely related to *P. asparagi*. The *P. niederhauseri* x *P. asparagi*-related hybrid nature of this isolate was confirmed by its dual *β-tubulin* sequence, while its rDNA ITS sequence was identical to that of *P. asparagi* (data not shown). This could be the result of concerted evolution or a side effect of hybridization. It shows once more that species identification and hybrid detection can fail if only ITS and/or mitochondrial loci are used.

Both clade 7c species *P. cinnamomi* and *P. parvispora*, are putative hybrids based on our GBS and FCM data. *Phytophthora cinnamomi* contained considerably more loci than *P. infestans*, also a heterothallic species, which would not be the case if it were an autopolyploid. The analysed isolates consist of four genotypes, with the ex-type strain of *P. cinnamomi* (PRI0393 = CBS144.22) being clearly distinct from the other isolates. The hybrid status of *P. cinnamomi* could explain why this species has an exceptionally wide host range (Zentmyer 1980) and causes such devastating disease in natural and horticultural environments (Burgess et al. 2017; Kamoun et al. 2015). *Phytophthora* hybrids indeed can display an expanded host range compared to their parental species and can potentially have an increased pathogenicity (Bertier et al. 2013; Érsek 1995; Gu and Ko 2001; Jafari et al. 2020; Man in ‘t Veld et al. 2007).

Extensive hybridization between several clade 8a species has been demonstrated previously (Safaiefarahani et al. 2016). We identified several *P. cryptogea* isolates as hybrids, based on the large number of loci and the shared loci with the *P.* ×*pseudocryptogea* hybrids and non-hybrid *P. cryptogea* isolates*.* Two of these hybrids were included in the study of Robideau et al. (2011), CBS114074 and CBS468.81, and have an ITS sequence similar to *P. cryptogea* but a *cox*I sequence identical to that of *P. pseudocryptogea*, which indicates that they are hybrids of *P. cryptogea* and *P. pseudocryptogea*. Also *P. drechsleri* and *P.* sp. kelmania are hybrids, probably between species that have yet to be identified. Just like *P. pseudocryptogea* × *P.* sp. kelmania, these hybrids contain a substantial proportion of triallelic loci, confirming they are the result of hybridization.

Our results of clade 8b are in line with the observations made by Bertier et al. (2013). They identified isolate CBS138.87 as a hybrid between *P. porri* and *P.* taxon parsley. The latter species is very closely related to *P. primulae* as it has identical ITS and *Ypt*1 sequences. This agrees with the high allele similarity we observed between this hybrid and *P. primulae*. The reported genome size of this hybrid (319 Mbp/2C; Bertier et al. 2013) is substantially larger than the diploid genome of *P. porri* and the genome of *P. primulae*, which is assumed to be a tetraploid species. Also, the number of loci is much higher than those of both these species. These results suggest it is an allopolyploid hybrid. The genome size of *P. primulae* agrees with that observed by Bertier et al. (2013). Because in this species nearly all loci are monoallelic, the large genome size is probably the result of genome duplication, suggesting it is an autopolyploid species.

### Hierarchical clustering

The classification of *Phytophthora* in multiple phylogenetic clades as previously determined (Blair et al. 2008; Cooke et al. 2000; Jung et al. 2017b; Yang et al. 2017) was largely corroborated by the hierarchical clustering. An exception was clade 8b, which clustered together with all clade 10 species, except *P. gemini*. The analyzed clade 8b species shared few loci with the other clade 8 species and even fewer with *Phytophthora* species from other clades (Table S4). Similar results were obtained for *Halophytophthora* and *Phytopythium* species. However, clade 8b clustered together with the other clade 8 species in the phylogenetic trees assessed by RAxML and ASTRAL-III on loci30 (Figure S5a and c). In all our phylogenetic trees, as well as in published trees (Blair et al. 2008; Kroon et al. 2004; Martin et al. 2014; Yang et al. 2017), the clade 8b branch lengths are exceptionally long, confirming that this subclade is genetically very distinct from the other clade 8 species. In addition, these species behave differently as they cause disease under cold conditions and mainly infect winter-grown vegetables (Redondo et al. 2018b). The separation of the clade 8b species *P. porri* and *P. primulae* could be a reflection of their primitive and *Pythium*-like combination of morphological characters, i.e. lateral and multiple, paragynous antheridia, and semipapillate, persistent, and intercalary sporangia (Brasier and Hansen 1992; Erwin and Ribeiro 1996). Clade 8d species *P. syringae* is less primitive, lacking the lateral and multiple antheridia and intercalary sporangia. Clade 8a species *P. cryptogea*, *P. drechsleri,* and *P. erythoseptica,* form amphigynous antheridia and terminal non-papillate sporangia (Erwin and Ribeiro 1996). Clade 8c species have terminal sporangia, which are exclusively (*P. hibernalis* and *P. ramorum*) or partly (*P. lateralis*) caducous as adaptation to a fully or partially aerial lifestyle, and have mostly amphigynous antheridia (Brasier et al. 2012; Erwin and Ribeiro 1996; Werres et al. 2001).

Another discrepancy was that clade 10 species *P. boehmeriae*, *P. kernoviae,* and *P. morindae,* were separated from *P. gallica* and were positioned in proximity of clade 8b and clade 4. *P. gallica* differs from the other clade 10 species in that it is sterile and produces non-papillate sporangia (Jung and Nechwatal 2008; Nelson and Abad 2010).

### Phylogenomic analysis

Since preliminary tests using phylogenetic network methods (e.g. PhyloNet) gave inconsistent or extremely reticulated results, we decided to leave out the putative hybrids and construct phylogenomic trees rather than networks.

As suggested by Dupuis et al. (2017) and Anderson et al. (2017), we used both a concatenation- and coalescent-based phylogenomic approach on two sets of selected loci (loci30 and loci80). Both methods generally gave higher statistical support when using loci30, between as well as within (sub)clades (Figure S5a and S5b). A higher support when a large proportion of the data is missing is in agreement with previous phylogenomic GBS studies (Anderson et al. 2017; Arbizu et al. 2016; Nute et al. 2018). It should be emphasized that in our case the missing data itself are true biological observations in the sense that they are the result of genetic divergence (SNPs in the restriction sites cause the loss of loci or a high number of SNPs causes the reads to be assigned to different loci; see Fig. 1a), and not merely the result of an unsaturated sequencing depth. The loci in loci80 were present in most species and thus represent a conserved fraction of the *Phytophthora* genome because the restriction sites were conserved and SNP divergence was low. Therefore, these loci are relatively uninformative, leading to low bootstrap support/posterior probabilities and wrong placement of some species and subclades (Figure S5c and d).

Topology largely agrees on the ASTRAL and concatenation tree in case of loci30, although some clades (4 and 12), subclades (2d and 2e, 8b and 8d, 9a2 and 9b) and species (*P. botryosa*, *P. mirabilis*, *P. cactorum*, *P. pseudotsugae*, *P. palmivora*, *P. pseudosyringae*, *P. amnicola*, *P. thermophila*, *P. pinifolia*, *P. chlamydospora*, *P. quininea,* and *P. richardiae*) are positioned differently. Agreement between two alternative phylogenomic methods depends on the dataset (Dupuis et al. 2017) and might be explained by reduced accuracy of summary methods such as ASTRAL when gene tree estimation error (GTEE) is high (Roch and Warnow 2015; Vachaspati and Warnow 2018). The short GBS sequences can indeed lead to high GTEE (Chou et al. 2015; Roch and Warnow 2015) and poorly informative gene trees (Dupuis et al. 2017; Fernández-Mazuecos et al. 2018). In that case, concatenation-based methods perform better, even when incomplete lineage sorting (ILS) is high (Chou et al. 2015; Hosner et al. 2016; Vachaspati and Warnow 2018). Higher statistical support in concatenation-based phylogenies may, however, mask real biological reasons for low support of branches such as ILS or hybridization (Anderson et al. 2017). Since we do not know the true species tree and gene trees, we also do not know the levels of ILS and GTEE in our dataset. Minimal incongruence between the two phylogenomic methods for closely related species suggests limited ILS, and we assume high GTEE because of short GBS loci.

The contrasting topology of both methods was mostly associated with a different placement of clades. This is probably the result of the very small number of shared loci among highly divergent species. Indeed, reduced representation methods such as RAD-seq or GBS are especially suitable for studying closely related species (Arbizu et al. 2016; DaCosta and Sorenson 2016; Leaché et al. 2015; Zimmer and Wen 2015). Nevertheless, we find similar interclade relationships as the ones observed in trees that were constructed based on protein data from complete genomes of 21 *Phytophthora* species from eight clades using Matrix representation using parsimony (MRP), gene tree parsimony (GTP) and a supermatrix of concatenated datasets (McCarthy and Fitzpatrick 2017). We equally obtain low statistical support for the close relationship between clades 1, 2 and 3 (mainly in the concatenation-based tree). While in the trees of McCarthy and Fitzpatrick (2017) clades 2 and 3 are sister clades (but with varying bootstrap support), in our trees clade 3 is basal to clades 1, 2, 4 and 12 (clades 4 and 12 were not included in their study). Also, clade 6 is a sister clade of clade 8 in their MRP and GTP phylogenies, albeit with limited support (bootstrap of 59 and 57, respectively). However, in their tree based on a supermatrix analysis, like in our trees, clade 6 is basal to all other clades except clade 10 (there were no clade 9 species included in their study). Applying GBS to a more comprehensive set of species per clade might help to further elucidate interclade and interspecies relationships in the *Phytophthora* phylogeny.

Positioning hybrids in a phylogeny can create problems, particulary because a reticulation process such as hybridization cannot be displayed by a simple bifurcating tree. Therefore, hybrids should be identified by the method we applied here and excluded from the phylogenetic analysis. Only after construction of a phylogenomic tree of non-hybrid species, hybrids can be anchored to (the branches of) their progenitors, without exerting their disturbing effects during the construction of the phylogenetic tree. In this way, we endeavoured to eliminate these hybrid-induced errors and produce a reliable phylogeny.

This study is yet another step in the exploration of the genus *Phytophthora*. Future studies with an expanded set of species, including additional ex-type strains, can provide new insights on genetic relationships in and between clades. GBS – separately or in combination with other techniques such as FCM – can also be applied to related oomycete or fungal genera for species identification, to assess the presence of hybrids or to elaborate their phylogeny.

Finally, our GBS data can be further explored and used to develop applications such as real-time PCR, hybridization probes, multiplex amplicon sequencing, etc. and may eventually lead to the development of (sub)clade-, species or genotype specific markers that can routinely be used for the identification of species and their hybrids.

## CONCLUSIONS

Our study sheds a new light on the occurrence of interspecific hybridization in *Phytophthora.* By applying GBS, in combination with genome size estimation, we were able to identify *Phytophthora* hybrids, including 16 new hybrid species and the discovery of the first interclade hybrid. Reliable recognition of hybrids is important for plant protection, as they can display an increased epidemiological risk compared to their progenitors. It is also indispensable for the correct treatment of hybrids in phylogenetic studies, as their positioning in phylogenetic tree can be cumbersome and lead to suboptimal tree topologies. By excluding the identified hybrids from phylogenomic analyses, we have constructed a reliable phylogenomic tree of the genus *Phytophthora*. We could also employ the GBS data for reliable species identification and reveal intraspecific genetic diversity at an unprecedented resolution.

## Supplementary Information

**Additional file 1: Table S1**. Details of the samples used in the study. Isolates indicated in bold are ex-type strains or authentic strains. From isolates marked with an asterisk only DNA was obtained.

**Additional file 2: Table S2**. Genome size of selected *Phytophthora* isolates as estimated by flow cytometry. Numbers in italics refer to the size of nuclei that correspond to secondary fluorescence peaks. Results from isolates in bold were adopted from Jung et al. (2017c). A dash in the column of standard deviation (SD) and coefficient of variation (CV) indicates the corresponding fluorescence peak was only encountered in one of the measurements.

**Additional file 3: Table S3**. Reproducibility of GBS loci identification, allele similarity and allele numbers of biological and technical replicates within and between runs.

**Additional file 4: Table S4**. Pairwise comparison of GBS loci of all isolates. See legend of Fig. 2 for details.

**Additional file 5: Table S5**. Combined similarity index (CSI) for each pair of non-hybrid isolates. Isolate codes in bold are ex-type strains or authentic strains (see Table S1 for details). The CSI values are colour-coded with dark green for values ≥80%, pale green for values between 55 and 80%, yellow for values between 50 and 55% and no colour for values ≤50%.

**Additional file 6 : Figure S1**. Histogram showing the distribution of the average read depth per locus per isolate. Percentage of the 1 762 508 loci with a read depth of at least 15, 20, and 25 is indicated in the graph.

**Additional file 7 : Figure S2**. Saturation curve analysis of reads after preprocessing. a) and b) saturation of the number of loci and the allele similarity for two diploid species (*P. cactorum* and *P. plurivora*), two diploid hybrids (*P. ×serendipita* and *P. ×pelgrandis*) and a polyploid hybrid (*P. ×heterohybrida*), calculated by comparing the number of loci and/or allele similarity of subsampled datasets at increasing numbers of reads against all reads from that sample. c) and d) saturation of the number of shared loci and the allele similarity between samples of the diploid progenitors *P. cactorum* and *P. hedraiandra* and between them and their hybrid *P. ×serendipita*, calculated by comparing the number of shared loci and/or allele similarity of subsampled datasets of the first sample at increasing numbers of reads against all reads of the second sample. e) and f) saturation of the number of shared loci and the allele similarity between samples of species with a more complex genome, i.e. polyploid hybrids *P. ×heterohybrida*, *P. ×incrassata* and the diploid species with a large genome size (330 Mbp) *P. uniformis*, calculated by comparing the number of shared loci and/or allele similarity of subsampled datasets of the first sample at increasing numbers of reads against all reads of the second sample.

**Additional file 8 : Figure S3**. a) Number of GBS loci with one to four alleles in isolates from *Phytophthora* clade 1. b) Number of GBS loci with one to four alleles in isolates from *Phytophthora* clade 2. c) Number of GBS loci with one to four alleles in isolates from *Phytophthora* clades 3, 4 and 5. d) Number of GBS loci with one to four alleles in isolates from *Phytophthora* clade 6. e) Number of GBS loci with one to four alleles in isolates from *Phytophthora* clade 7. f) Number of GBS loci with one to four alleles in isolates from *Phytophthora* clade 8. g) Number of GBS loci with one to four alleles in isolates from *Phytophthora* clades 9, 10 and 12.

**Additional file 9 : Figure S4**. Hierarchical clustering of the binary (absence/presence) GBS locus data constructed using supraHEX with average linkage and 500 bootstrap replicates. Numbers on branches indicate bootstrap values.

**Additional file 10 : Figure S5**. a) Concatenation-based phylogenomic tree using RAxML on 61111 SNPs from 1610 loci that occur in 30% of a set of representative *Phytophthora* isolates from all clades. Numbers on branches indicate bootstrap values. b) Concatenation-based phylogenomic tree using RAxML on 1062 SNPs from 29 loci that occur in 80% of a set of representative *Phytophthora* isolates of all clades. Numbers on branches indicate bootstrap values. c) Coalescence-based phylogenomic tree using ASTRALIII on 61111 SNPs from 1610 loci that occur in 30% of a set of representative *Phytophthora* isolates of all clades. Numbers on branches indicate bootstrap values. d) Coalescence-based phylogenomic tree using ASTRALIII on 1062 SNPs from 29 loci that occur in 80% of a set of representative *Phytophthora* isolates of all clades. Numbers on branches indicate bootstrap values.

**Additional file 11 : Figure S6**. Concatenation-based phylogenomic tree using RAxML on 61111 SNPs from 1610 loci that occur in 30% of a set of representative *Phytophthora* isolates of all clades, with hybrids anchored to their progenitors. Numbers on branches indicate bootstrap values. Hybrids are indicated in bold, positioned separately and linked to the parental species. If a parental species is unknown, the accolade points to the longest branch of the subclade to which the parental species belongs. If the genotype of the parental species is unknown, accolades are placed at the branch of the tree in which the parental species reside.

## Data Availability

The datasets generated and analysed during the current study are available in the Dryad Digital Repository, at 10.5061/dryad.p40r314. All scripts that were used in the GBS data analysis are distributed under the MIT license and are available in a repository called “GBS_Phytophthora” v.1.0.0, and can be downloaded from GitLab (https://gitlab.com/ahaegeman/GBS_Phytophthora) and Zenodo with doi: 10.5281/zenodo.3363287.
