# Supplementary material for: Unravelling hybridization in Phytophthora using phylogenomics and genome size estimation
Source: IMA Fungus. 2021 Jul 1;12:16. doi: 10.1186/s43008-021-00068-w (PMC8246709; doi:10.1186/s43008-021-00068-w)
Supplement: Supplementary file 2 — Additional file 2: Table S2. Genome size of selected Phytophthora isolates as estimated by flow cytometry. Numbers in italics refer to the size of nuclei that correspond to secondary fluorescence peaks. Results from isolates in bold were adopted from Jung et al. (2017c). A dash in the column of standard deviation (SD) and coefficient of variation (CV) indicates the corresponding fluorescence peak was only encountered in one of the measurements. [file 43008_2021_68_MOESM2_ESM.pdf]

Table S2

Genome size of selected Phytophthora isolates as estimated by flow cytometry. Numbers in italics refer to the size of nuclei that correspond to secondary fluorescence peaks. Results from isolates in bold were adopted from Jung et al. (2017). A dash in the column of standard deviation (SD) and coefficient of variation (CV) indicates the corresponding fluorescence peak was only encountered in one of the measurements.

clade	species	abbre-viation	isolate code	average (Mbp/2C)	SD (Mbp/2C)	average (pg/2C)	SD (pg/2C)	CV (%)
1a	*Phytophthora cactorum*	CAC	05/005	188	9	0.192	0.009	4.6%
			05/008	182	5	0.186	0.005	2.6%
			06/002	178	3	0.182	0.003	1.8%
	P. hedraiandra	HED	06/018	189	5	0.193	0.005	2.4%
			06/019	186	7	0.190	0.007	3.6%
	P. ×serendipita	×SER	05/009	185	2	0.189	0.002	1.2%
			05/012	184	9	0.188	0.009	4.9%
			08/005	178	5	0.182	0.005	2.9%
			10/013	180	3	0.184	0.003	1.6%
			HC21	175	4	0.179	0.005	2.6%
			HC25	192	2	0.196	0.002	1.0%
			HC27	180	6	0.184	0.006	3.2%
			HC32	186	2	0.190	0.003	1.3%
			HC39	178	10	0.181	0.010	5.7%
			HC42	179	6	0.183	0.006	3.1%
			HC50	184	3	0.188	0.003	1.5%
			W54	185	4	0.189	0.004	2.3%
1	*P. nicotianae*	NIC	13/006	189	17	0.193	0.018	9.1%
			13/036	185	7	0.189	0.007	3.7%
			13/048	178	3	0.182	0.003	1.8%
1-1a	*P. ×pelgrandis*	×PEL	15/006	186	2	0.190	0.002	1.0%
			15/007	119	12	0.122	0.012	9.7%
			15/008	187	7	0.191	0.007	3.7%
			15/009	180	2	0.184	0.002	1.1%
1b	*P. tentaculata*	TEN	SW22	260	7	0.265	0.007	2.6%
			SW23	258	13	0.263	0.013	5.0%
1c	*P. infestans*	INF	16/053	545	14	0.558	0.015	2.6%
			17/033	*147*	-	*0.150*	-	-
				317	5	0.324	0.005	1.4%
				515	*10*	*0.527*	*0.010*	*1.9%*
				841	*5*	*0.860*	*0.005*	*0.5%*
			17/034	546	4	0.558	0.004	0.7%
			JA07	780	15	0.797	0.015	1.9%
2a	*P. botryosa*	BOT	TJ092	181	5	0.185	0.005	2.6%
	P. meadii	MEA	TJ095	175	9	0.179	0.009	4.9%
	P. citrophthora-related	CIPr	TJ097	155	3	0.158	0.003	1.6%
	P. ×citrophthora-related1	×CIPr1	TJ100	124	2	0.127	0.002	1.9%
			TJ101	152	1	0.155	0.001	0.5%
			TJ102	176	5	0.180	0.005	2.7%
			TJ103	194	6	0.199	0.006	2.9%

	P. ×*citrophthora*-related2	×CIPr2	TJ093	147	6	0.150	0.006	3.8%
	P. ×*citrophthora*1	×CIP1	TJ104	125	1	0.128	0.001	0.9%
	P. ×*citrophthora*2	×CIP2	TJ184	156	4	0.160	0.004	2.6%
	P. citrophthora	CIP	10/083	131	6	0.134	0.006	4.4%
			10/087	125	3	0.128	0.003	2.6%
	P. occultans	OCC	05/034	129	4	0.132	0.004	2.8%
			12/021	132	6	0.135	0.006	4.4%
			13/037	123	4	0.126	0.005	3.7%
	P. terminalis	TER	10/099	131	3	0.134	0.003	2.2%
2b	*P. tropicalis*	TRO	SW11	112	6	0.115	0.006	4.9%
			TJ099	128	1	0.131	0.001	0.8%
2c	*P. acerina*	ACE	14/013	120	0	0.123	0.000	0.3%
	P. multivora	MUV	10/014	124	1	0.127	0.001	0.5%
			10/055	119	3	0.121	0.004	2.9%
	P. pini	PIN	10/030	122	2	0.125	0.003	2.0%
			14/002	119	8	0.122	0.008	6.4%
	P. plurivora	PLV	10/033	124	1	0.127	0.001	0.6%
			10/034	123	4	0.125	0.004	3.4%
			11/010	122	3	0.125	0.003	2.6%
			11/014	125	3	0.128	0.003	2.5%
			12/023	119	3	0.122	0.003	2.2%
			14/057	112	11	0.115	0.011	10.0%
			14/065	179	6	0.183	0.006	3.2%
3	*P. pseudosyringae*	PSR	SW17	157	5	0.160	0.005	3.1%
			SW18	157	14	0.160	0.015	9.1%
			AC10	150	4	0.154	0.005	2.9%
			AC11	154	10	0.158	0.010	6.5%
4	*P. quercetorum*	QCT	TJ024	138	6	0.141	0.006	4.3%
			TJ025	145	2	0.149	0.002	1.1%
5	*P. heveae*	HEVr	TJ013	137	3	0.140	0.003	2.0%
			TJ014	129	3	0.132	0.003	1.9%
			TJ016	142	4	0.145	0.004	2.7%
	P. castaneae	CAS	TJ033	136	3	0.139	0.003	2.2%
			TJ034	149	4	0.153	0.004	2.4%
6a	*P.* taxon Walnut	WAL	SW33	129	3	0.132	0.003	2.1%
	P. inundata	INU	09/001	303	3	0.310	0.004	1.1%
			09/003	295	9	0.302	0.009	2.9%
6b	*P. thermophila* × *amnicola*	THE×AMN	TJ114	159	2	0.163	0.002	1.3%
			TJ115	146	1	0.149	0.001	0.7%
	P. amnicola × *chlamydospora*	AMN×CHL	TJ118	157	3	0.161	0.004	2.2%
			TJ119	165	2	0.169	0.002	1.0%
	P. chlamydospora	CHL	04/001	150	8	0.153	0.008	5.4%
			04/004	152	10	0.155	0.010	6.5%
			07/003	152	13	0.155	0.013	8.3%
			10/003	154	8	0.157	0.008	5.4%
			12/040	142	17	0.145	0.018	12.1%
	P. chlamydospora × *lacustris*	CHL×LAC	13/005	159	9	0.163	0.009	5.8%

			SS01	225	26	0.230	0.026	11.4%
	P. lacustris	LAC	05/013	173	1	0.177	0.001	0.6%
			07/002	153	17	0.157	0.017	10.8%
			CH01	166	3	0.169	0.003	1.5%
			CH04	158	5	0.161	0.005	3.4%
	P. gonapodyides	GON	05/004	171	11	0.175	0.011	6.3%
			05/015	178	10	0.182	0.010	5.4%
			05/017	164	6	0.167	0.006	3.8%
			05/025	274	7	0.281	0.008	2.7%
			06/001	266	6	0.272	0.006	2.3%
			07/004	272	10	0.278	0.011	3.9%
			07/007	274	8	0.280	0.009	3.1%
			14/049	176	7	0.180	0.007	3.9%
	P. megasperma	MEG	14/085	283	7	0.290	0.007	2.6%
	P. crassamura	CRA	TJ179	218	15	0.223	0.015	6.9%
			TJ180	217	4	0.222	0.004	1.9%
7a	*P. ×alni*	×ALN	AC01	532	6	0.544	0.006	1.2%
			AC02	513	30	0.525	0.030	5.8%
			TJ145	505	12	0.517	0.012	2.3%
			TJ143	565	10	0.578	0.010	1.8%
			AC03	844	32	0.863	0.033	3.8%
	P. ×multiformis	×MUF	AC04	653	13	0.668	0.013	1.9%
	P. uniformis	UNI	AC05	348	9	0.356	0.009	2.7%
			AC06	317	5	0.324	0.005	1.5%
			AC07	327	3	0.335	0.003	0.8%
	P. ×cambivora	×CAM	05/006	458	9	0.469	0.009	1.9%
			05/027	472	6	0.483	0.006	1.3%
			10/010	429	16	0.439	0.017	3.8%
			**AC08**	**426**		**0.436**		
			**AC09**	**510**		**0.521**		
			**AC12**	**423**		**0.433**		
			**AC13**	**428**		**0.438**		
			**TJ067**	**462**		**0.472**		
			**TJ068**	**445**		**0.455**		
			**TJ069**	**458**		**0.468**		
			**TJ070**	**445**		**0.455**		
			**TJ071**	**435**		**0.445**		
			**TJ072**	**460**		**0.470**		
			**TJ073**	**285**		**0.291**		
			**TJ074**	***297***		***0.304***		
				**457**		**0.467**		
			**TJ076**	**457**		**0.467**		
			**TJ077**	**462**		**0.472**		
			**TJ078**	**468**		**0.479**		
			**TJ081**	**489**		**0.500**		
			**TJ083**	**480**		**0.491**		
			**TJ085**	**478**		**0.489**		
			**TJ086**	**458**		**0.468**		
			**TJ087**	**451**		**0.461**		

			**TJ090**	**447**		**0.457**		
	P. ×*cambivora*-related	×CAMr	**TJ075**	**479**		**0.490**		
			**TJ079**	**454**		**0.464**		
			**TJ080**	***315***		***0.322***		
				**395**		**0.404**		
	P. ×*heterohybrida*	×HET	**TJ036**	**338**		**0.346**		
			**TJ037**	**324**		**0.331**		
			**TJ038**	**331**		**0.338**		
			**TJ039**	**339**		**0.347**		
			**TJ040**	**343**		**0.351**		
			**TJ041**	**327**		**0.334**		
			**TJ042**	**330**		**0.337**		
			**TJ043**	**329**		**0.336**		
			**TJ046**	**340**		**0.348**		
			**TJ047**	**320**		**0.327**		
			**TJ048**	**318**		**0.325**		
			**TJ049**	**313**		**0.320**		
			**TJ050**	**334**		**0.342**		
			**TJ053**	**339**		**0.347**		
			**TJ055**	**320**		**0.327**		
			**TJ057**	**323**		**0.330**		
	P. ×*heterohybrida*-related	×HETr	TJ106	543	12	0.556	0.012	2.2%
			TJ107	518	17	0.530	0.018	3.3%
			TJ108	561	11	0.574	0.011	1.9%
			TJ109	541	5	0.553	0.005	0.9%
	P. ×*incrassata*	×INC	**TJ060**	**619**		**0.633**		
			**TJ061**	***421***		***0.430***		
				**639**		**0.653**		
			**TJ062**	***426***		***0.436***		
				**648**		**0.663**		
			**TJ063**	**650**		**0.665**		
			**TJ064**	**654**		**0.669**		
			**TJ065**	**651**		**0.666**		
			**TJ066**	**648**		**0.663**		
	P. fragariae	FRA	JA06	281	7	0.288	0.007	2.3%
			SW39	273	8	0.279	0.008	2.8%
	P. europaea	EUR	SW37	206	3	0.211	0.003	1.5%
	P. ×*attenuata*-related	×ATTr	TJ105	301	14	0.307	0.014	4.5%
7b	*P. niederhauseri*	NIE	AB02	325	5	0.332	0.005	1.6%
			AB04	343	4	0.351	0.004	1.1%
			SW15	350	2	0.358	0.002	0.6%
	P. niederhauseri-related	NIEr	AB05	403	17	0.413	0.017	4.1%
7c	*P. parvispora*	PAS	SW45	220	2	0.225	0.002	0.7%
	P. cinnamomi	CIN	10/009	406	19	0.416	0.020	4.8%
			11/024	410	4	0.419	0.005	1.1%
			14/003	400	16	0.409	0.016	4.0%
8a	*P. cryptogea*	CRY	SW34	217	4	0.222	0.004	2.0%
			05/022	216	8	0.221	0.008	3.5%
			12/016	219	7	0.224	0.007	3.1%

			12/025	220	3	0.225	0.003	1.5%
			14/011	224	7	0.229	0.008	3.3%
8b	*P. primulae*	PRI	SW47	255	1	0.261	0.001	0.4%
8c	*P. lateralis*	LAT	13/001	156	1	0.159	0.001	0.8%
			JA03	154	6	0.157	0.006	3.8%
			JA04	146	4	0.149	0.004	2.5%
	P. hibernalis	HIB	SW43	215	2	0.219	0.002	0.7%
	P. ramorum	RAM	02/001	141	6	0.144	0.006	4.1%
			02/002	134	7	0.137	0.007	5.3%
			11/038	140	9	0.144	0.009	6.1%
			14/004	134	10	0.137	0.010	7.5%
8d	*P. syringae*	SYR	TJ021	171	3	0.175	0.003	1.7%
			TJ022	173	3	0.177	0.003	1.7%
			04/005	184	16	0.188	0.017	8.9%
9a1	*P. hydropathica*	HYD	TJ018	136	2	0.139	0.002	1.4%
	P. sp. Peru4-related	PER4r	TJ019	136	4	0.139	0.004	2.9%
9a3	*P. polonica*	POL	SW16	126	2	0.128	0.002	1.7%
			TS01	120	0	0.123	0.000	0.3%
10	*P. gallica*	GAL	SW13	144	2	0.147	0.002	1.6%
	P. kernoviae	KER	06/003	147	10	0.150	0.010	6.5%
			10/022	132	6	0.134	0.006	4.8%
			12/006	112	8	0.115	0.008	7.0%
12	*P. quercina*	QCN	SW19	184	1	0.188	0.001	0.3%
			TJ027	181	3	0.185	0.003	1.5%
			TJ030	*93*	*2*	*0.095*	*0.002*	*2.3%*
				185	2	0.189	0.002	1.0%
			TJ196	183	4	0.187	0.004	2.1%
