# Supplementary material for: Unravelling hybridization in Phytophthora using phylogenomics and genome size estimation
Source: IMA Fungus. 2021 Jul 1;12:16. doi: 10.1186/s43008-021-00068-w (PMC8246709; doi:10.1186/s43008-021-00068-w)
Supplement: Supplementary file 3 — Additional file 3: Table S3. Reproducibility of GBS loci identification, allele similarity and allele numbers of biological and technical replicates within and between runs. [file 43008_2021_68_MOESM3_ESM.pdf]

Table S3

Reproducibility of GBS loci identification, allele similarity and allele numbers of biological and technical replicates within and between runs.

	isolate[a]	species	abbreviation	replicates	DNA[b]	number of loci		allele similarity (%)[d]	number of alleles[e]		
						mean	SD[c]		shared	unique	% shared
intra-run	02/001	*P. ramorum*	RAM	2	=	20942	66	99.99	26986	532	98.1
	10/045	*P. cactorum*	CAC	3	=	15746	43	100.00 (0.00)	15997 (42)	95 (42)	99.4 (0.3)
	10/051	*P. hedraiandra*	HED	2	=	16047	54	100.00	16379	147	99.1
	10/114	*P. porri*	POR	2	≠	10976	110	99.99	11414	559	95.3
	11/013	*P. cactorum*	CAC	2	≠	15707	100	99.99	15909	211	98.7
	12/006	*P. kernoviae*	KER	2	≠	12678	12	100.00	12808	63	99.5
	15/008	*P. ×pelgrandis*	×PEL	3	=	27868	432	99.99 (0.01)	29589 (328)	1359 (225)	95.6 (0.7)
	CBS109229	*P. ipomoeae*	IPO	3	≠	18390	188	99.98 (0.00)	20805 (184)	985 (197)	95.5 (0.9)
	CBS581.69	*P. botryosa*	BOT	2	≠	12097	7	99.94	14373	1135	92.7
	HC21	*P. ×serendipita*	×SER	3	≠	19251	150	99.99 (0.01)	26039 (146)	653 (121)	97.6 (0.5)
	SW14	*P. niederhauseri*	NIE	2	≠	30712	177	99.94	38940	3341	92.1
	TJ001	*P. ×alni*	×ALN	2	≠	43617	139	99.90	60189	7671	88.7
	TJ008	*P. uniformis*	UNI	2	≠	23022	74	100.00	24352	590	97.6
	TJ027	*P. quercina*	QUE	2	≠	18541	13	100.00	18816	260	98.6
	TJ091	*P. botryosa*	BOT	2	≠	12206	15	99.99	14974	151	99.0
inter-run	02/001	*P. ramorum*	RAM	5	=	21014	63	99.99 (0.01)	26115 (103)	500 (87)	98.1 (0.3)
	10/022	*P. kernoviae*	KER	2	=	12641	46	100.00	12740	27	99.1
	14/011	*P. cryptogea*	CRY	2	≠	23572	127	99.99	31036	931	94.8
	15/007	*P. ×pelgrandis*	×PEL	2	≠	26136	996	99.98	26427	1835	86.0
	15/053	*P. cactorum*	CAC	3	≠	15653	109	100.00 (0.01)	15855 (105)	117 (103)	98.8 (0.6)
	16/053	*P. infestans*	INF	2	≠	18819	10	99.93	22306	1163	90.9
	AC10	*P. pseudosyringae*	PSR	2	=	21843	156	100.00	22044	149	98.5

AC14	*P. plurivora*	PLV	2	=	15660	66	99.99	15789	44	99.2
AC16	*P. pseudosyringae*	PSR	2	=	22133	109	99.99	22405	48	98.9
AC22	*P.* sp. hungarica	HUN	2	=	36365	606	99.99	42259	396	97.0
AC24	*P. megasperma*	MEG	2	=	32123	1719	99.97	47016	1948	91.2
CBS129.23	*P. cryptogea*	CRY	2	≠	17069	441	99.96	19502	1129	91.0
CBS200.81	*P. humicola*	HUM	2	=	24575	20	99.99	32176	246	98.8
CBS235.30/TJ098	*P. citrophthora*-related	CIPr	2	≠	12675	391	100.00	15820	575	94.6
CBS292.35/SW30	*P. drechsleri*	DRE	2	≠	24531	704	99.89	31958	3136	82.9
CBS411.96/SW45	*P. parvispora*	PAS	2	≠	30598	150	99.98	36881	370	97.4
JA06/SW38	*P. fragariae*	FRA	2	≠	22505	14	100.00	23732	127	99.2
JA07	*P. infestans*	INF	2	≠	19225	132	99.95	23410	1678	87.7
SW31	*P. melonis*	MEL	2	≠	29091	11	99.99	31387	260	97.5
TJ021	*P. syringae*	SYR	2	≠	20553	16	100.00	21284	61	99.3
TJ035	*P. castaneae*	CAS	2	=	16276	38	99.99	16704	94	99.0
TJ099	*P. tropicalis*	TRO	2	≠	15342	238	99.99	20302	704	94.8
TJ103	*P.* ×*citrophthora*-related1	×CIPr1	2	≠	14941	313	99.99	19801	758	94.4
TJ190	*P. pseudosyringae*	PSR	2	≠	22032	93	99.99	22299	50	99.1

[a] two codes separated by a slash indicate identical isolates with a different isolate code (see Table S1)
[b] ≠ : DNA from independent extractions was used for library preparation; = : the same DNA-extract was used for library preparation
[c] SD: standard deviation
[d] mean value in case of >2 replicates with standard deviation between brackets
[e] mean value in case of >2 replicates with standard deviation between brackets; shared = shared alleles between replicates; unique = unique alleles for each replicate
