# Supplementary material for: Unravelling hybridization in Phytophthora using phylogenomics and genome size estimation
Source: IMA Fungus. 2021 Jul 1;12:16. doi: 10.1186/s43008-021-00068-w (PMC8246709; doi:10.1186/s43008-021-00068-w)
Supplement: Supplementary file 6 — Additional file 6 : Figure S1. Histogram showing the distribution of the average read depth per locus per isolate. Percentage of the 1 762 508 loci with a read depth of at least 15, 20, and 25 is indicated in the graph. [file 43008_2021_68_MOESM6_ESM.pdf]

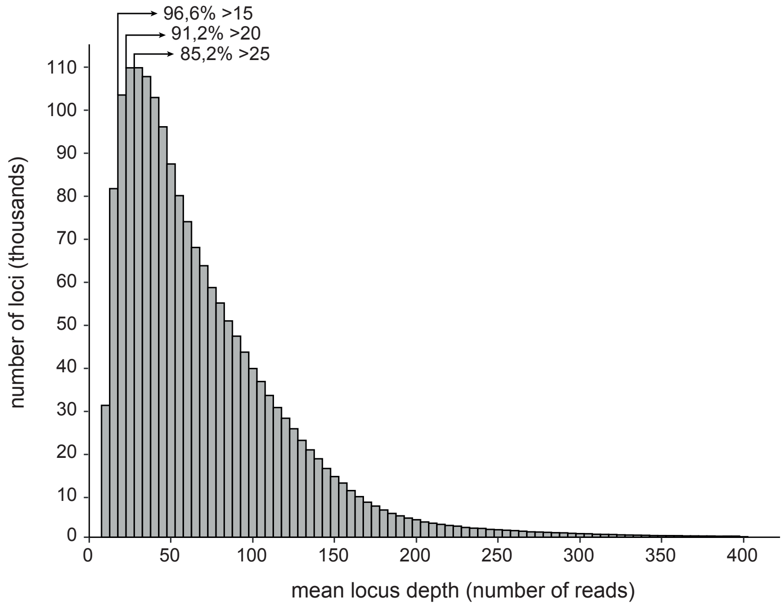

Figure S1
Histogram showing the distribution of the average read depth per locus per isolate.
Percentage of the 1 762 508 loci with a read depth of at least 15, 20, and 25 is indicated in the graph.
