# Supplementary material for: Unravelling hybridization in Phytophthora using phylogenomics and genome size estimation
Source: IMA Fungus. 2021 Jul 1;12:16. doi: 10.1186/s43008-021-00068-w (PMC8246709; doi:10.1186/s43008-021-00068-w)
Supplement: Supplementary file 7 — Additional file 7 : Figure S2. Saturation curve analysis of reads after preprocessing. a) and b) saturation of the number of loci and the allele similarity for two diploid species (P. cactorum and P. plurivora), two diploid hybrids (P. ×serendipita and P. ×pelgrandis) and a polyploid hybrid (P. ×heterohybrida), calculated by comparing the number of loci and/or allele similarity of subsampled datasets at increasing numbers of reads against all reads from that sample. c) and d) saturation of the number of shared loci and the allele similarity between samples of the diploid progenitors P. cactorum and P. hedraiandra and between them and their hybrid P. ×serendipita, calculated by comparing the number of shared loci and/or allele similarity of subsampled datasets of the first sample at increasing numbers of reads against all reads of the second sample. e) and f) saturation of the number of shared loci and the allele similarity between samples of species with a more complex genome, i.e. polyploid hybrids P. ×heterohybrida, P. ×incrassata and the diploid species with a large genome size (330 Mbp) P. uniformis, calculated by comparing the number of shared loci and/or allele similarity of subsampled datasets of the first sample at increasing numbers of reads against all reads of the second sample. [file 43008_2021_68_MOESM7_ESM.pdf]

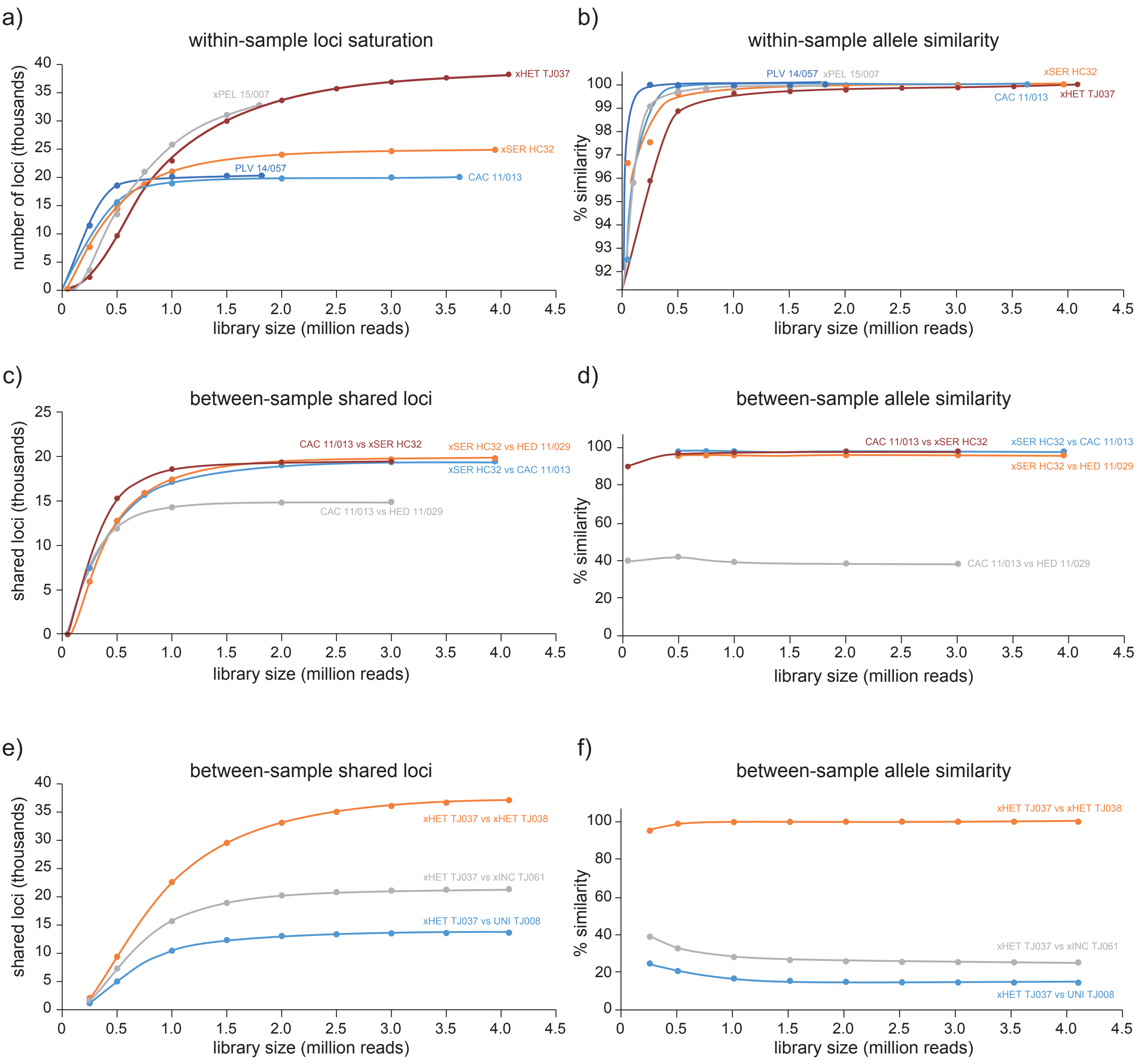

Figure S2

Saturation curve analysis of reads after preprocessing.

a) and b) saturation of the number of loci and the allele similarity for two diploid species (*P. cactorum* and *P. plurivora*), two diploid hybrids (*P. ×serendipita* and *P. ×pelgrandis*) and a polyploid hybrid (*P. ×heterohybrida*), calculated by comparing the number of loci and/or allele similarity of subsampled datasets at increasing numbers of reads against all reads from that sample.

c) and d) saturation of the number of shared loci and the allele similarity between samples of the diploid progenitors *P. cactorum* and *P. hedraiandra* and between them and their hybrid *P. ×serendipita*, calculated by comparing the number of shared loci and/or allele similarity of subsampled datasets of the first sample at increasing numbers of reads against all reads of the second sample.

e) and f) saturation of the number of shared loci and the allele similarity between samples of species with a more complex genome, i.e. polyploid hybrids *P. ×heterohybrida*, *P. ×incrassata* and the diploid species with a large genome size (330 Mbp) *P. uniformis*, calculated by comparing the number of shared loci and/or allele similarity of subsampled datasets of the first sample at increasing numbers of reads against all reads of the second sample.
