# Supplementary material for: Unravelling hybridization in Phytophthora using phylogenomics and genome size estimation
Source: IMA Fungus. 2021 Jul 1;12:16. doi: 10.1186/s43008-021-00068-w (PMC8246709; doi:10.1186/s43008-021-00068-w)
Supplement: Supplementary file 8 — Additional file 8 : Figure S3. a) Number of GBS loci with one to four alleles in isolates from Phytophthora clade 1. b) Number of GBS loci with one to four alleles in isolates from Phytophthora clade 2. c) Number of GBS loci with one to four alleles in isolates from Phytophthora clades 3, 4 and 5. d) Number of GBS loci with one to four alleles in isolates from Phytophthora clade 6. e) Number of GBS loci with one to four alleles in isolates from Phytophthora clade 7. f) Number of GBS loci with one to four alleles in isolates from Phytophthora clade 8. g) Number of GBS loci with one to four alleles in isolates from Phytophthora clades 9, 10 and 12. [file 43008_2021_68_MOESM8_ESM.pdf]

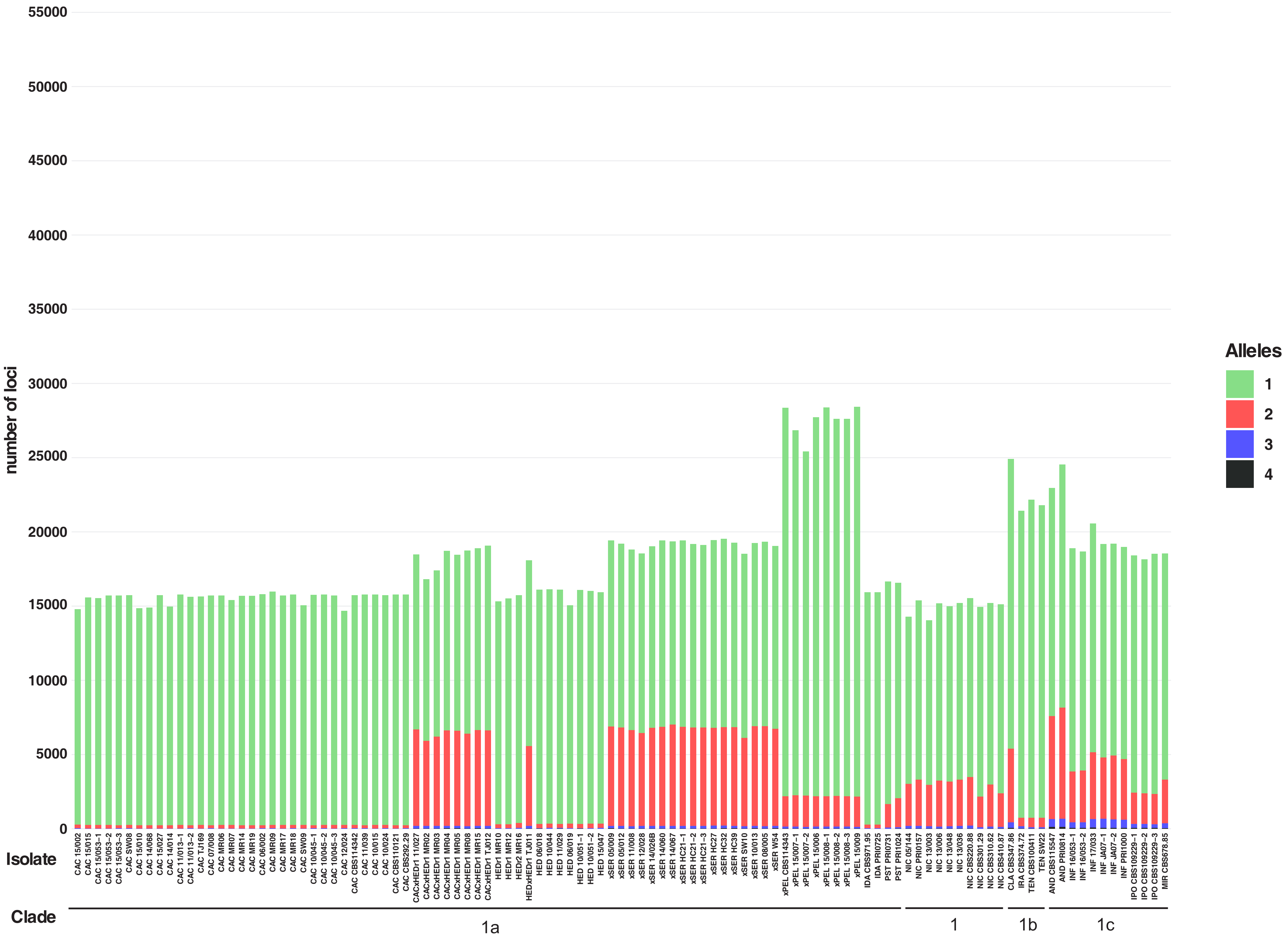

Figure S3a Number of GBS loci with one to four alleles in isolates from *Phytophthora* clade 1.

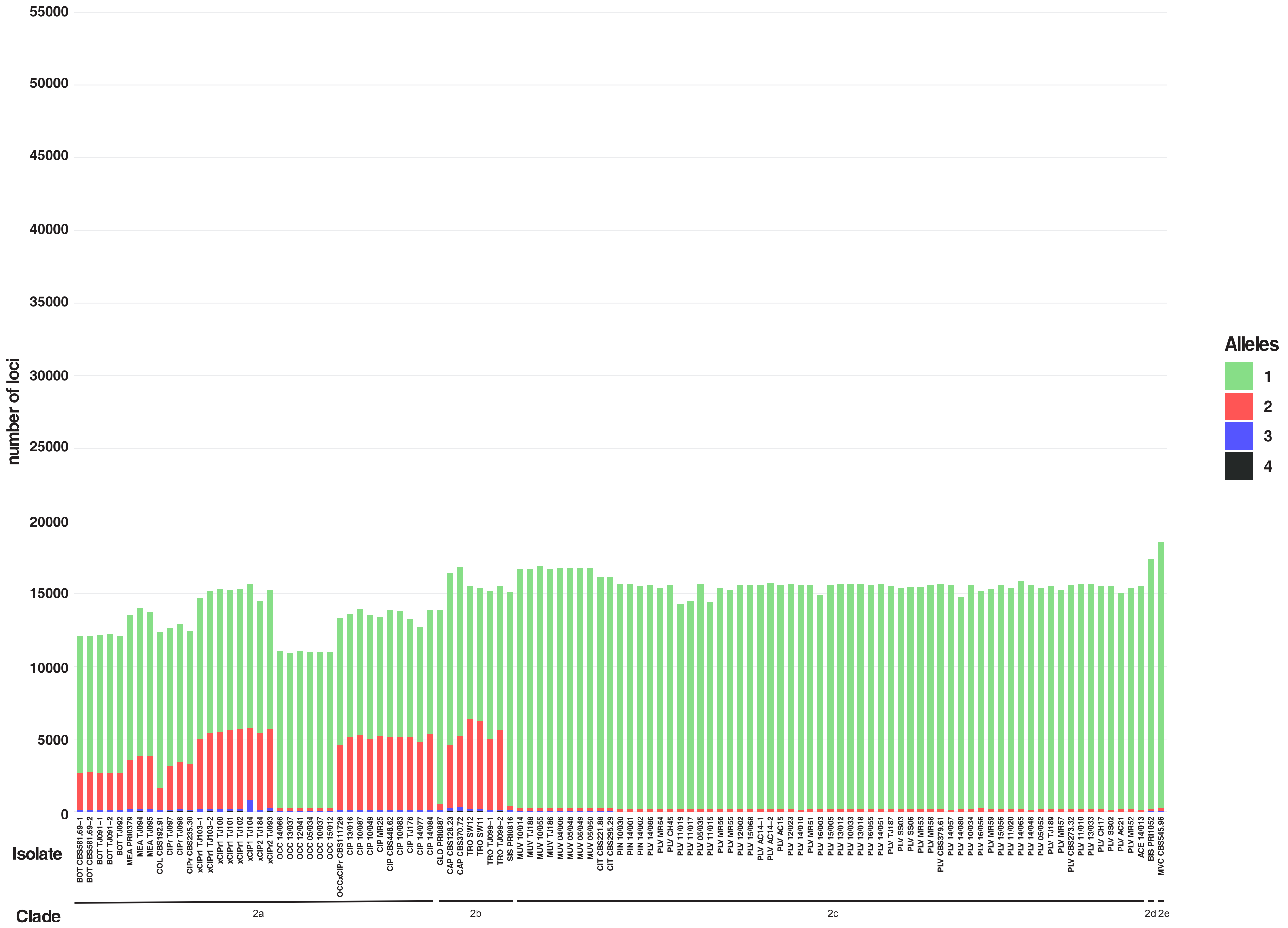

Figure S3b Number of GBS loci with one to four alleles in isolates from *Phytophthora* clade 2.

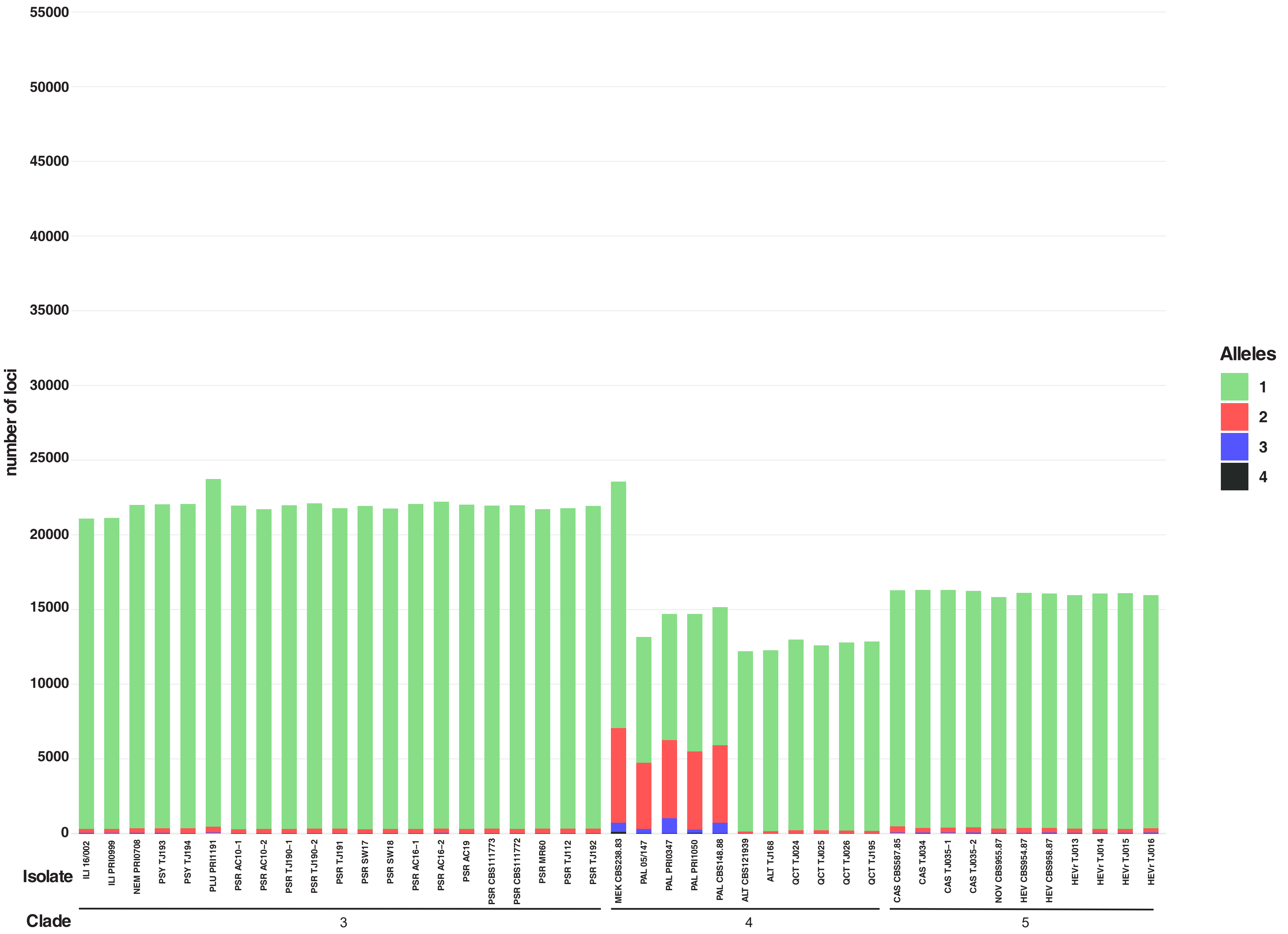

Figure S3c Number of GBS loci with one to four alleles in isolates from *Phytophthora* clades 3, 4 and 5.

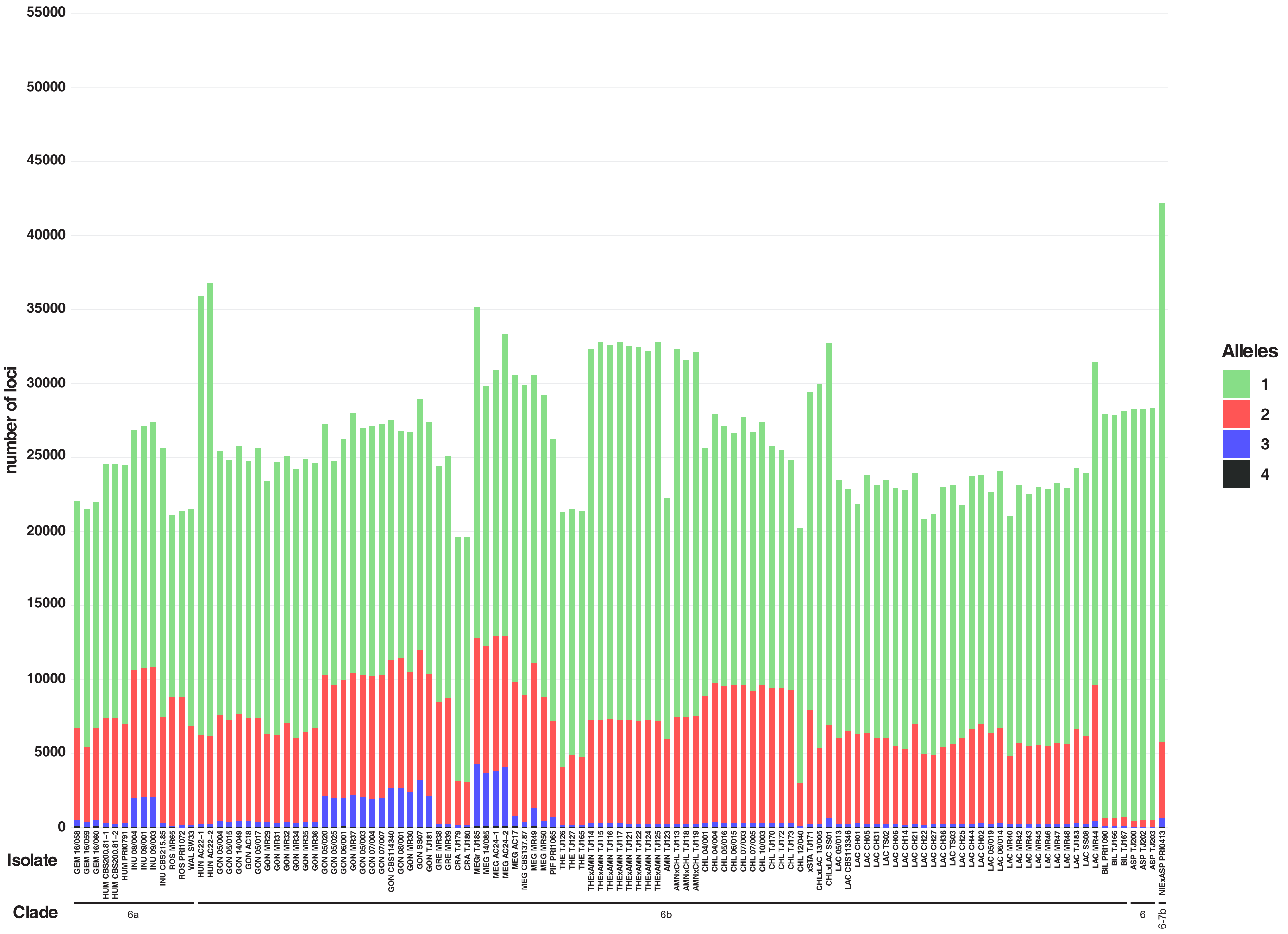

Figure S3d Number of GBS loci with one to four alleles in isolates from *Phytophthora* clade 6.

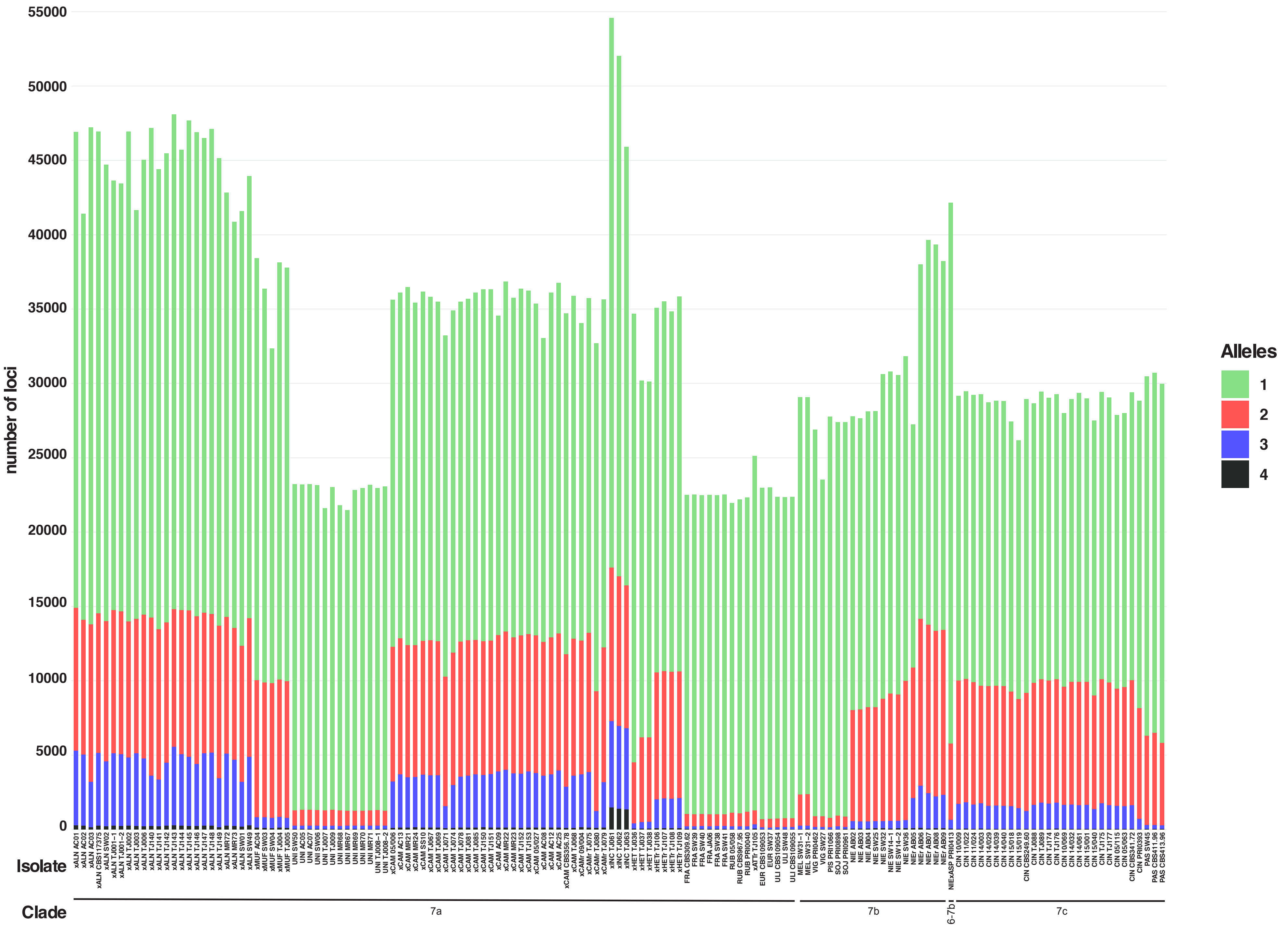

Figure S3e Number of GBS loci with one to four alleles in isolates from *Phytophthora* clade 7.

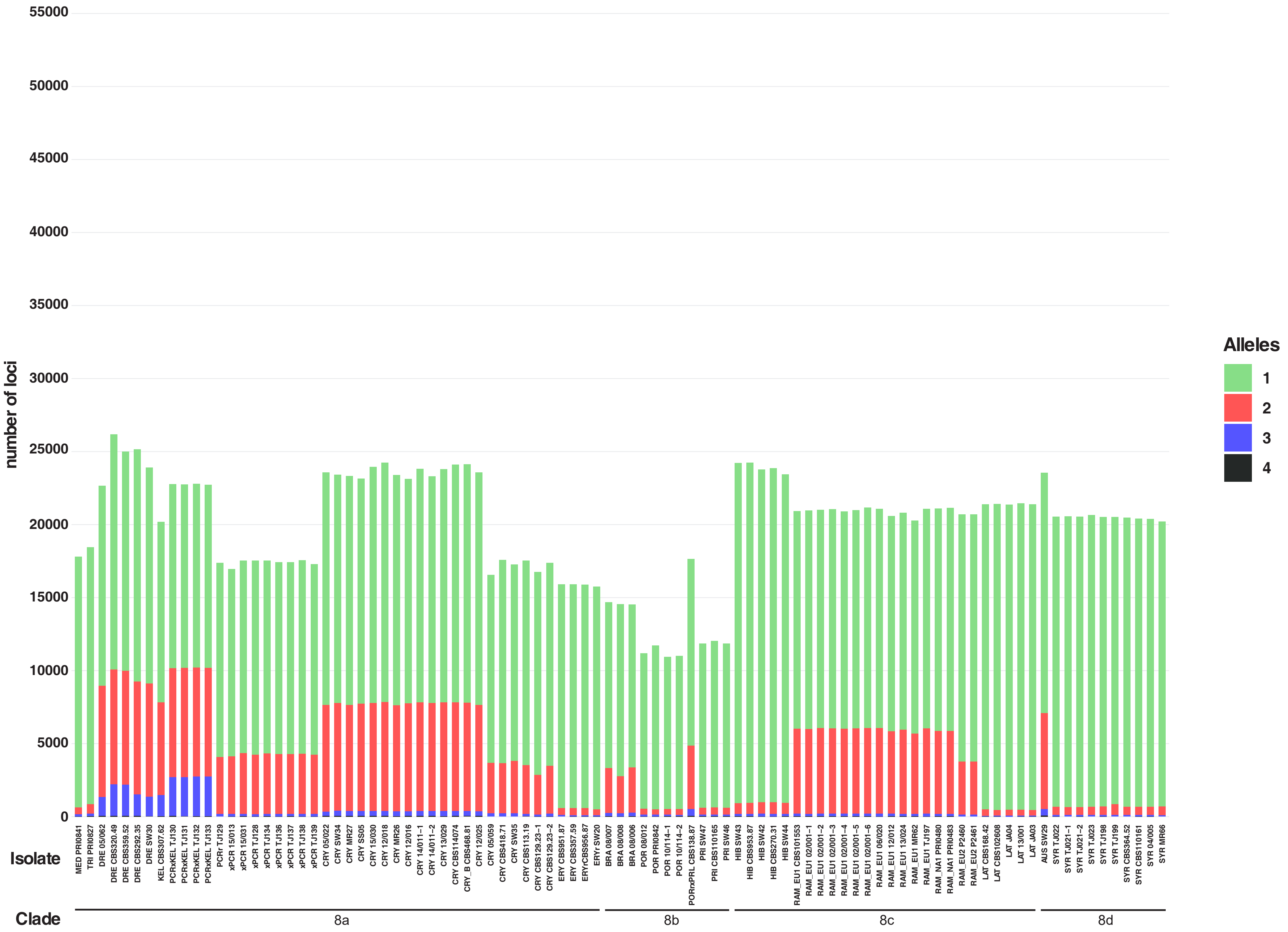

Figure S3f Number of GBS loci with one to four alleles in isolates from *Phytophthora* clade 8.

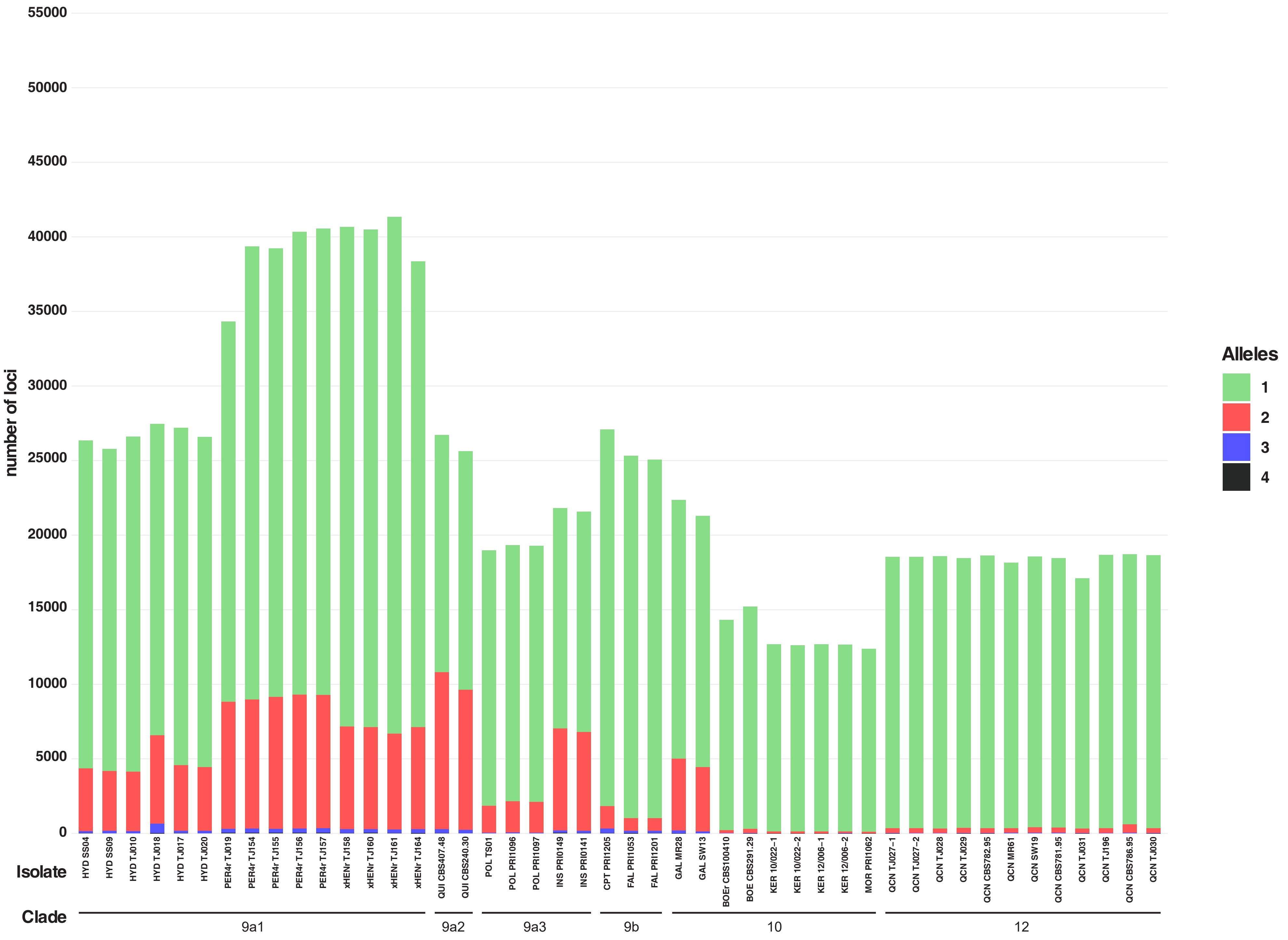

Figure S3g Number of GBS loci with one to four alleles in isolates from *Phytophthora* clades 9, 10 and 12.
