# Supplementary material for: Unravelling hybridization in Phytophthora using phylogenomics and genome size estimation
Source: IMA Fungus. 2021 Jul 1;12:16. doi: 10.1186/s43008-021-00068-w (PMC8246709; doi:10.1186/s43008-021-00068-w)
Supplement: Supplementary file 9 — Additional file 9 : Figure S4. Hierarchical clustering of the binary (absence/presence) GBS locus data constructed using supraHEX with average linkage and 500 bootstrap replicates. Numbers on branches indicate bootstrap values. [file 43008_2021_68_MOESM9_ESM.pdf]

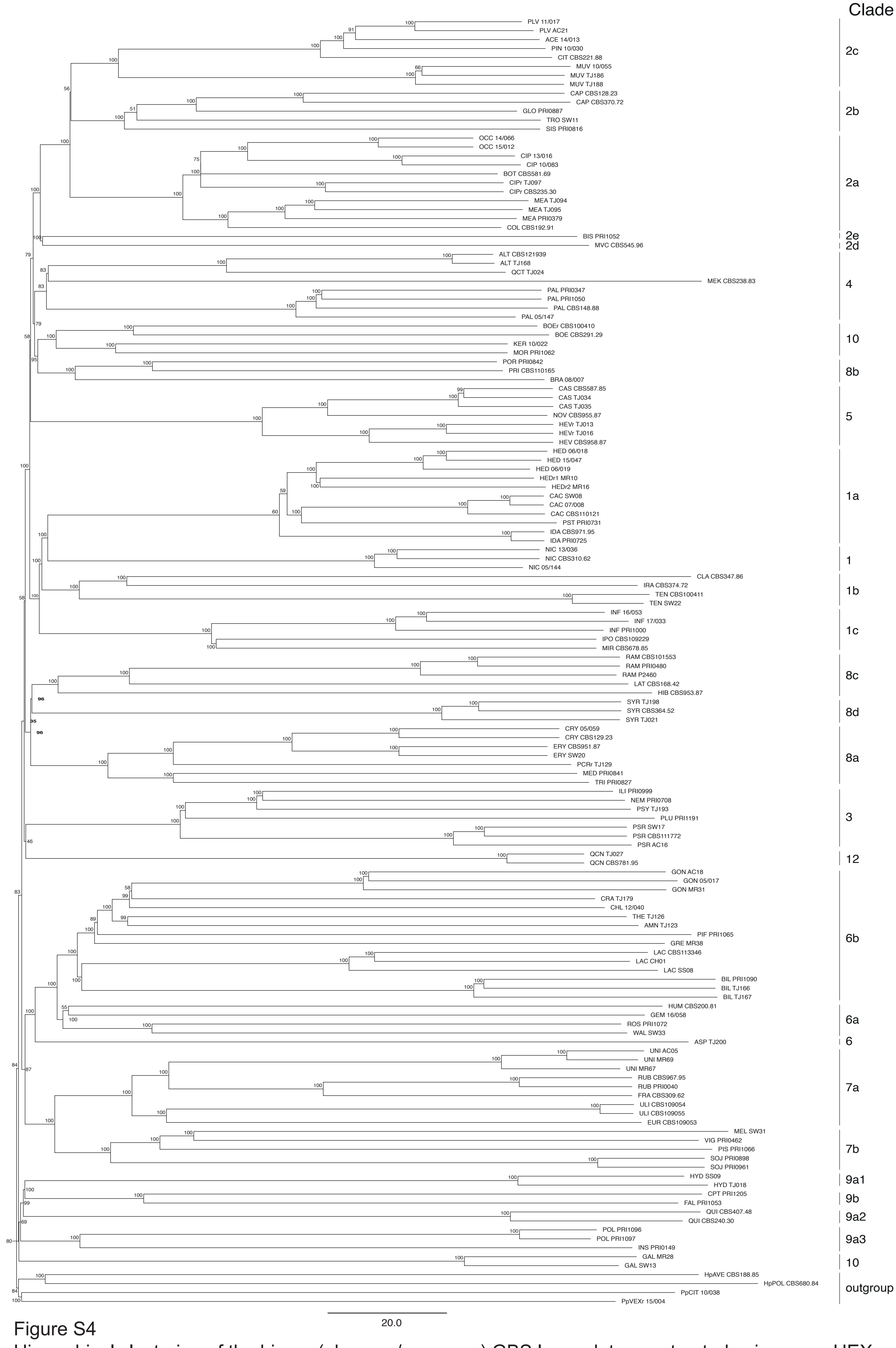

Figure S4

Hierarchical clustering of the binary (absence/presence) GBS locus data constructed using supraHEX with average linkage and 500 bootstrap replicates. Numbers on branches indicate bootstrap values.
