# Supplementary material for: Unravelling hybridization in Phytophthora using phylogenomics and genome size estimation
Source: IMA Fungus. 2021 Jul 1;12:16. doi: 10.1186/s43008-021-00068-w (PMC8246709; doi:10.1186/s43008-021-00068-w)
Supplement: Supplementary file 10 — Additional file 10 : Figure S5. a) Concatenation-based phylogenomic tree using RAxML on 61111 SNPs from 1610 loci that occur in 30% of a set of representative Phytophthora isolates from all clades. Numbers on branches indicate bootstrap values. b) Concatenation-based phylogenomic tree using RAxML on 1062 SNPs from 29 loci that occur in 80% of a set of representative Phytophthora isolates of all clades. Numbers on branches indicate bootstrap values. c) Coalescence-based phylogenomic tree using ASTRALIII on 61111 SNPs from 1610 loci that occur in 30% of a set of representative Phytophthora isolates of all clades. Numbers on branches indicate bootstrap values. d) Coalescence-based phylogenomic tree using ASTRALIII on 1062 SNPs from 29 loci that occur in 80% of a set of representative Phytophthora isolates of all clades. Numbers on branches indicate bootstrap values. [file 43008_2021_68_MOESM10_ESM.pdf]

Figure S5a (see legend below figure)

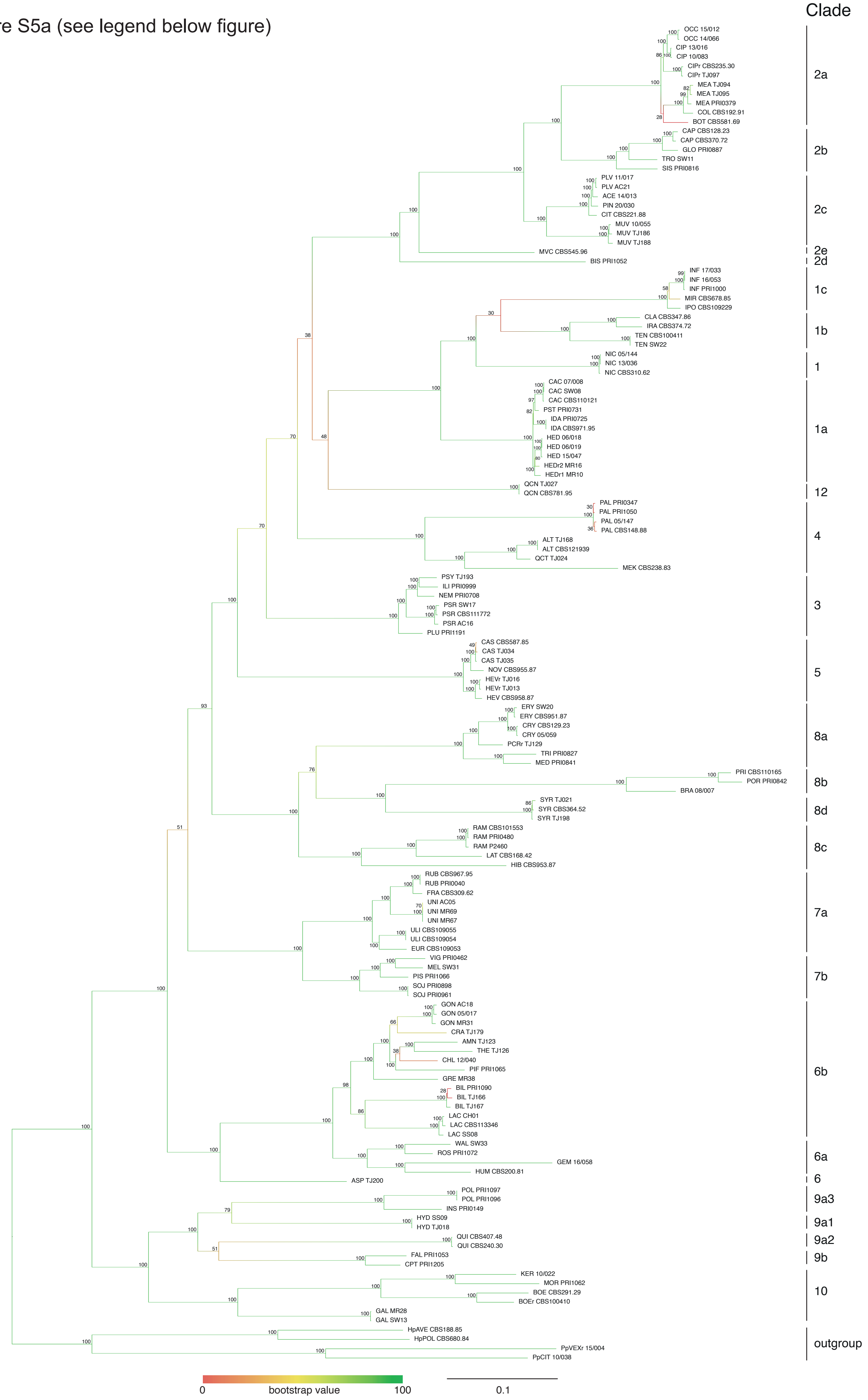

Figure S5a

Concatenation-based phylogenomic tree using RAxML on 61111 SNPs from 1610 loci that occur in 30% of a set of representative *Phytophthora* isolates from all clades. Numbers on branches indicate bootstrap values.

Figure S5b (see legend below figure)

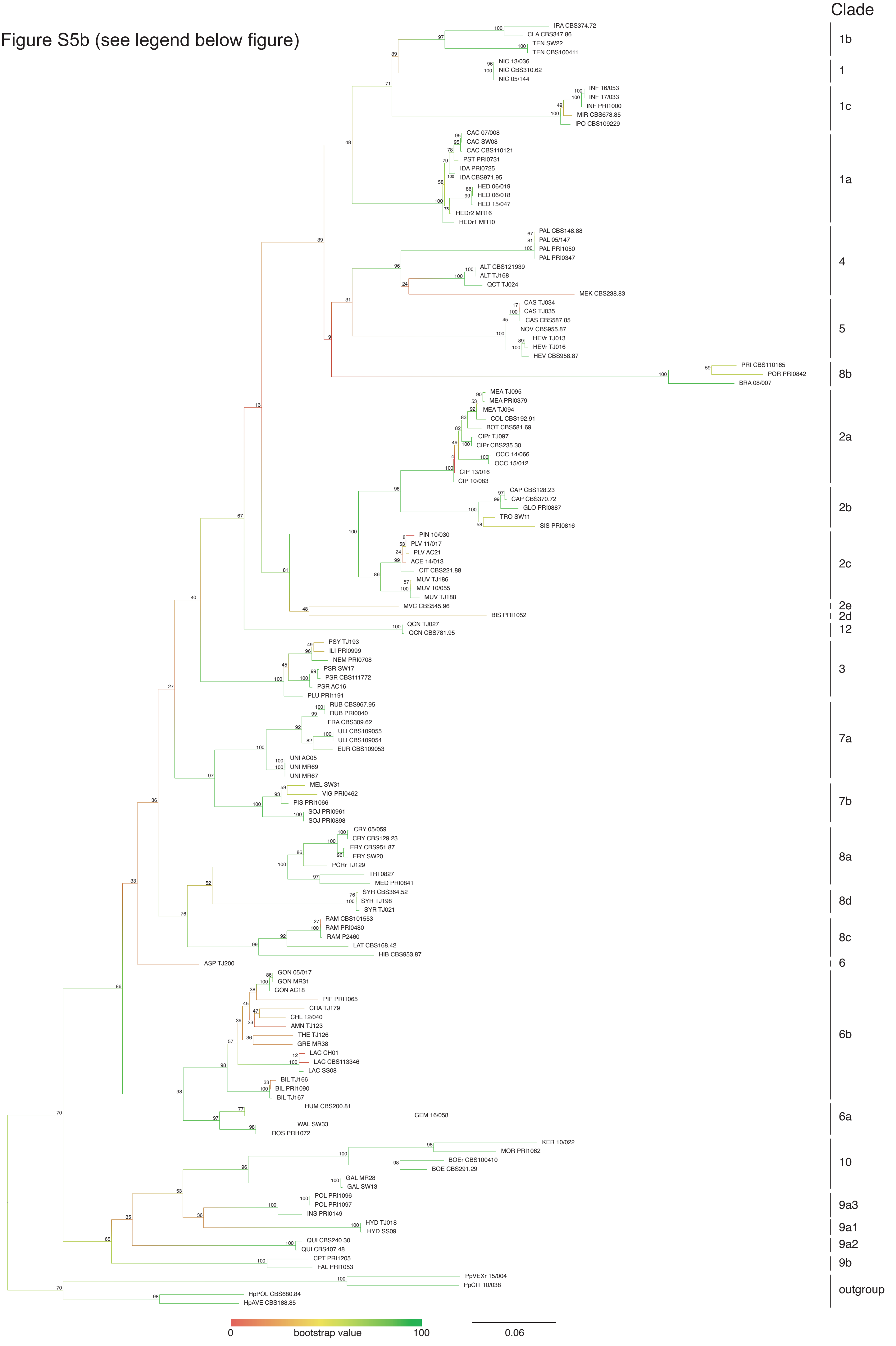

Figure S5b

Concatenation-based phylogenomic tree using RAxML on 1062 SNPs from 29 loci that occur in 80% of a set of representative *Phytophthora* isolates of all clades. Numbers on branches indicate bootstrap values.

Figure S5c (see legend below figure)

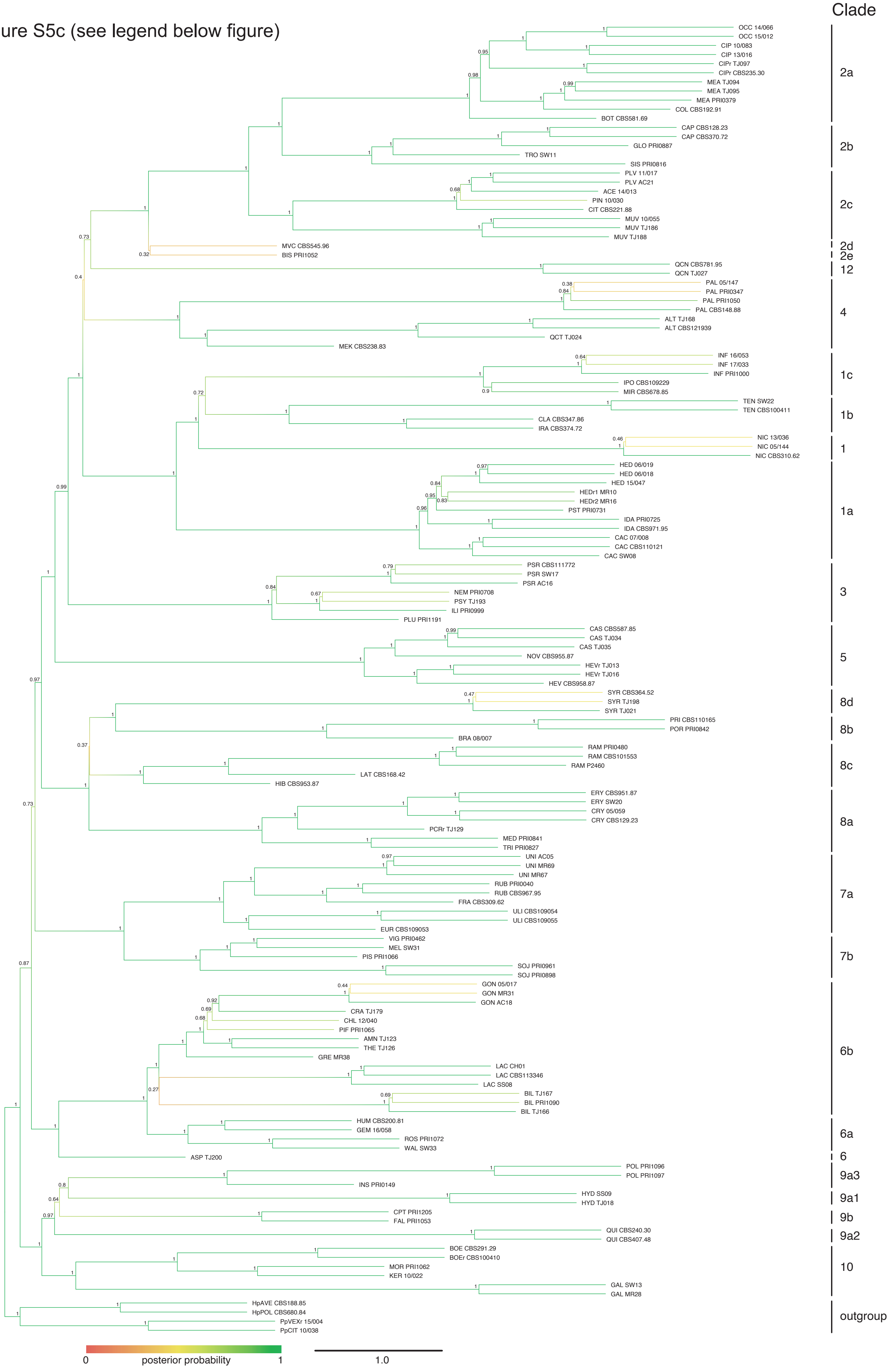

Figure S5c

Coalescence-based phylogenomic tree using ASTRALIII on 61111 SNPs from 1610 loci that occur in 30% of a set of representative *Phytophthora* isolates of all clades. Numbers on branches indicate bootstrap values.

Figure S5d (see legend below figure)

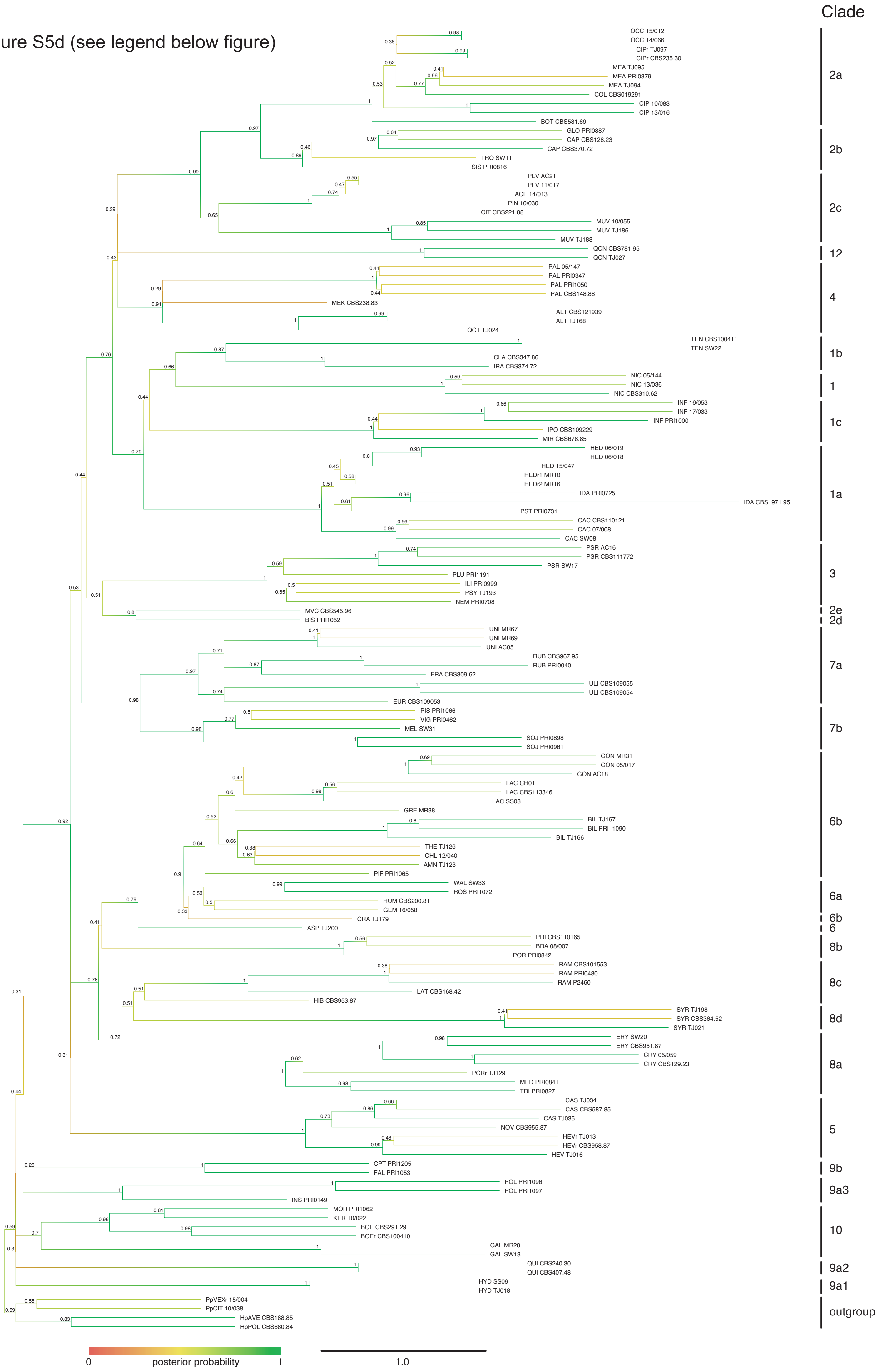

Figure S5d

Coalescence-based phylogenomic tree using ASTRALIII on 1062 SNPs from 29 loci that occur in 80% of a set of representative *Phytophthora* isolates of all clades. Numbers on branches indicate bootstrap values.
