# Supplementary material for: Unravelling hybridization in Phytophthora using phylogenomics and genome size estimation
Source: IMA Fungus. 2021 Jul 1;12:16. doi: 10.1186/s43008-021-00068-w (PMC8246709; doi:10.1186/s43008-021-00068-w)
Supplement: Supplementary file 11 — Additional file 11 : Figure S6. Concatenation-based phylogenomic tree using RAxML on 61111 SNPs from 1610 loci that occur in 30% of a set of representative Phytophthora isolates of all clades, with hybrids anchored to their progenitors. Numbers on branches indicate bootstrap values. Hybrids are indicated in bold, positioned separately and linked to the parental species. If a parental species is unknown, the accolade points to the longest branch of the subclade to which the parental species belongs. If the genotype of the parental species is unknown, accolades are placed at the branch of the tree in which the parental species reside. [file 43008_2021_68_MOESM11_ESM.pdf]

Figure S6 (see legend below figure)

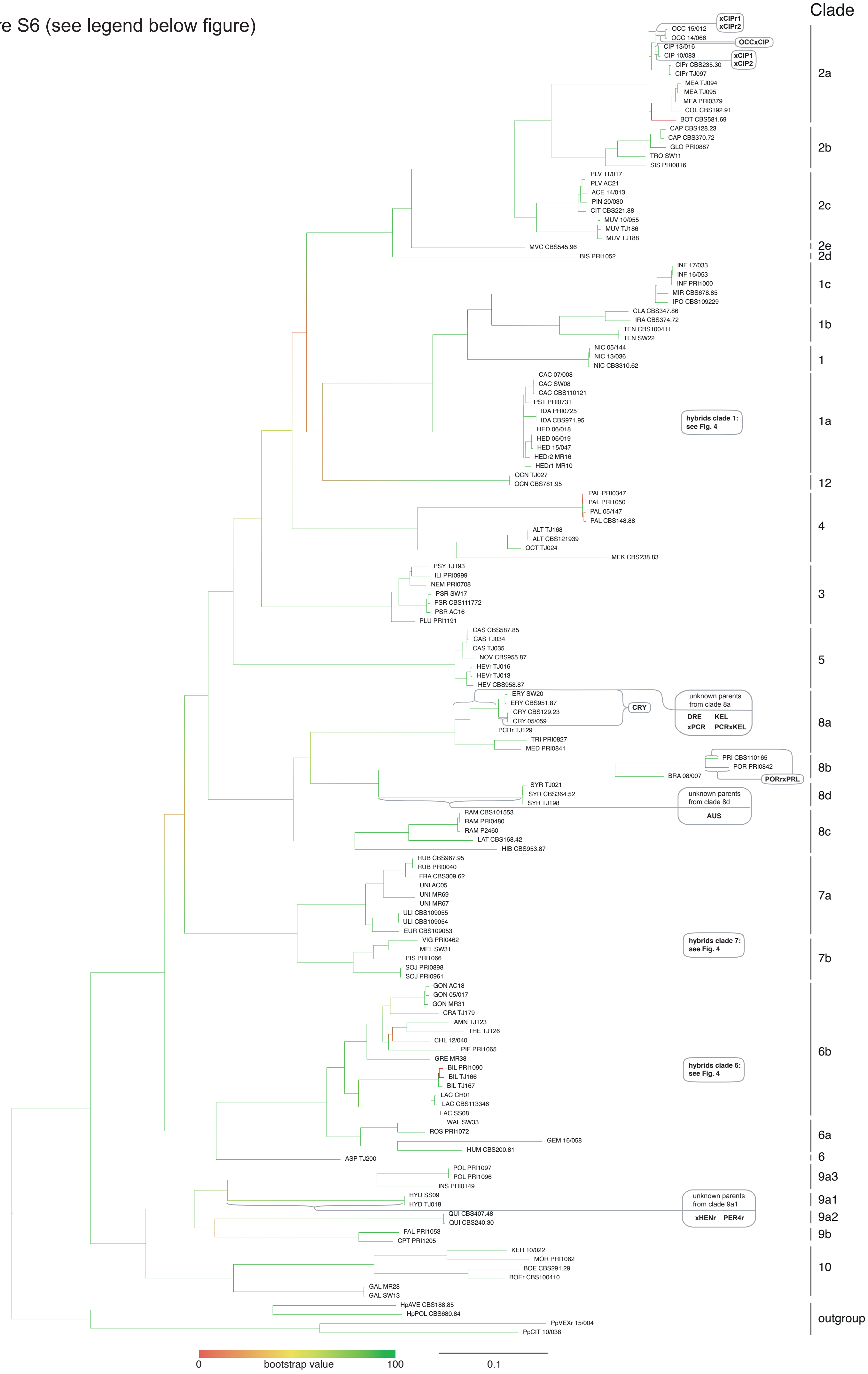

Figure S6

Concatenation-based phylogenomic tree using RAxML on 61111 SNPs from 1610 loci that occur in 30% of a set of representative *Phytophthora* isolates of all clades, with hybrids anchored to their progenitors. Numbers on branches indicate bootstrap values. Hybrids are indicated in bold, positioned separately and linked to the parental species. If a parental species is unknown, the accolade points to the longest branch of the subclade to which the parental species belongs. If the genotype of the parental species is unknown, accolades are placed at the branch of the tree in which the parental species reside.
